# Advanced Functional Electromagnetic Shielding Materials: A Review Based on Micro-Nano Structure Interface Control of Biomass Cell Walls

**DOI:** 10.1007/s40820-024-01494-2

**Published:** 2024-09-20

**Authors:** Yang Shi, Mingjun Wu, Shengbo Ge, Jianzhang Li, Anoud Saud Alshammari, Jing Luo, Mohammed A. Amin, Hua Qiu, Jinxuan Jiang, Yazeed M. Asiri, Runzhou Huang, Hua Hou, Zeinhom M. El-Bahy, Zhanhu Guo, Chong Jia, Kaimeng Xu, Xiangmeng Chen

**Affiliations:** 1 Co-Innovation Center of Efficient Processing and Utilization of Forest Resources, College of Materials Science and Engineering, Nanjing Forestry University, Nanjing, 210037 People’s Republic of China; 2 State Key Laboratory of Efficient Production of Forest Resourced, Beijing Forestry University, Qinghua East Road 35, Haidian District, Beijing, 100083 People’s Republic of China; 3 Department of Physics, Faculty of Sciences-Arar, Northern Border University, Arar, 91431 Saudi Arabia; 4 Department of Chemistry, College of Science, Taif University, P.O. Box 11099, 21944 Taif, Saudi Arabia; 5 Shaanxi Key Laboratory of Macromolecular Science and Technology, School of Chemistry and Chemical Engineering, Northwestern Polytechnical University, Xi’an, Shaanxi 710072 People’s Republic of China; 6 Integrated Composites Lab, Department of Mechanical and Construction Engineering, Northumbria University, Newcastle Upon Tyne, NE1 8ST UK; 7 College of Materials Science and Engineering, Taiyuan University of Science and Technology, Taiyuan, 030024 People’s Republic of China; 8 Department of Chemistry, Faculty of Science, Al-Azhar University, Nasr City, Cairo 11884 Egypt; 9 Yunnan Provincial Key Laboratory of Wood Adhesives and Glued Products, International Joint Research Center for Biomass Materials, Southwest Forestry University, Kunming, 650224 People’s Republic of China; 10 School of Science, Henan Agricultural University, Zhengzhou, 450002 People’s Republic of China

**Keywords:** Biomass materials, Electromagnetic interference shielding, Micro-nano structure interface control, Conductivity

## Abstract

Research efforts on electromagnetic interference (EMI) shielding materials have begun to converge on green and sustainable biomass materials. These materials offer numerous advantages such as being lightweight, porous, and hierarchical. Due to their porous nature, interfacial compatibility, and electrical conductivity, biomass materials hold significant potential as EMI shielding materials. Despite concerted efforts on the EMI shielding of biomass materials have been reported, this research area is still relatively new compared to traditional EMI shielding materials. In particular, a more comprehensive study and summary of the factors influencing biomass EMI shielding materials including the pore structure adjustment, preparation process, and micro-control would be valuable. The preparation methods and characteristics of wood, bamboo, cellulose and lignin in EMI shielding field are critically discussed in this paper, and similar biomass EMI materials are summarized and analyzed. The composite methods and fillers of various biomass materials were reviewed. this paper also highlights the mechanism of EMI shielding as well as existing prospects and challenges for development trends in this field.

## Highlights

- The advantages of biomass materials for electromagnetic interference (EMI) shielding are analyzed, the mechanism of EMI shielding is summarized, and the factors affecting EMI shielding are analyzed systematically.
- Various biomass materials (wood, bamboo, lignin, cellulose) were modified to obtain unique structures and improve EMI shielding performance.
- The problems encountered in the application of biomass materials for EMI shielding are summarized, and the potential development and application in the future are prospected.

## Graphical abstract

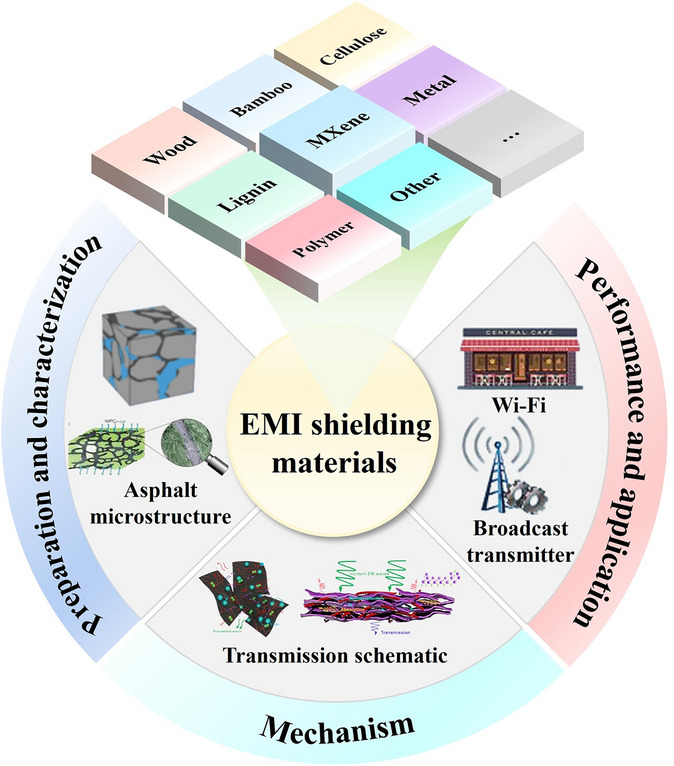

## Introduction

**Fig. 1:**
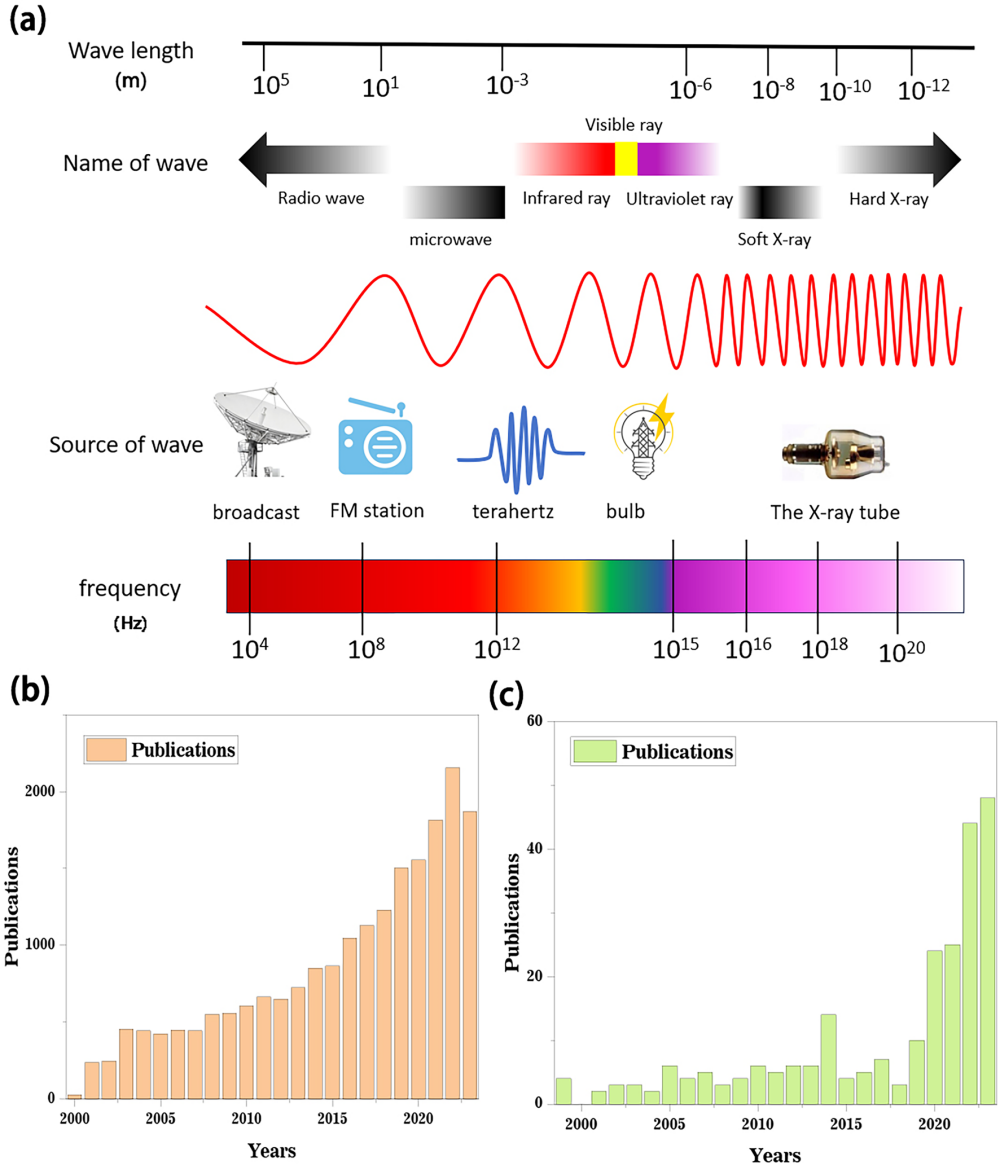
**a** Objects that often cause electromagnetic waves in life. **b** Articles published on electromagnetic shielding in the past few years. **c** Articles published on electromagnetic shielding of biomass in the two decades

The universal practice of mobile phones [1–3], computers [4], and other electronic devices [5–11] has transformed human society with unprecedented convenience [12–15]. However, this convenience comes at a cost, as these electronic devices are also responsible for electromagnetic interference (EMI) and pollution [16–20]. In fact, The World Health Organization (WHO) has listed electromagnetic radiation as the fourth largest source of environmental pollution after water pollution, air pollution and noise pollution. This escalating issue has sparked significant public concern [21]. It was found that electromagnetic pollution can obstruct the normal functioning of electronic equipment which would lead to malfunctions and potential data leakage. Additionally, it poses significant health risks to individuals such as headaches, insomnia, and lethargy [22, 23]. Therefore, it is crucial to prioritize the evolution of materials with efficient electromagnetic shielding to alleviate these risks while maintaining their properties for respective applications [24–27]. Figure 1a–c shows the potential source of electromagnetic waves in daily life and relevant studies published in the past few years. In recent years, the research on EMI shielding materials has gradually increased, but there are still relatively few studies on biomass EMI shielding materials. With people's attention to electromagnetic pollution and environment, biomass EMI materials have been studied relatively more in the past two years. This allows us to see the prospect of biomass EMI shielding materials, this paper will introduce the current biomass EMI shielding materials preparation, characteristics for the reference of researchers.

**Fig. 2:**
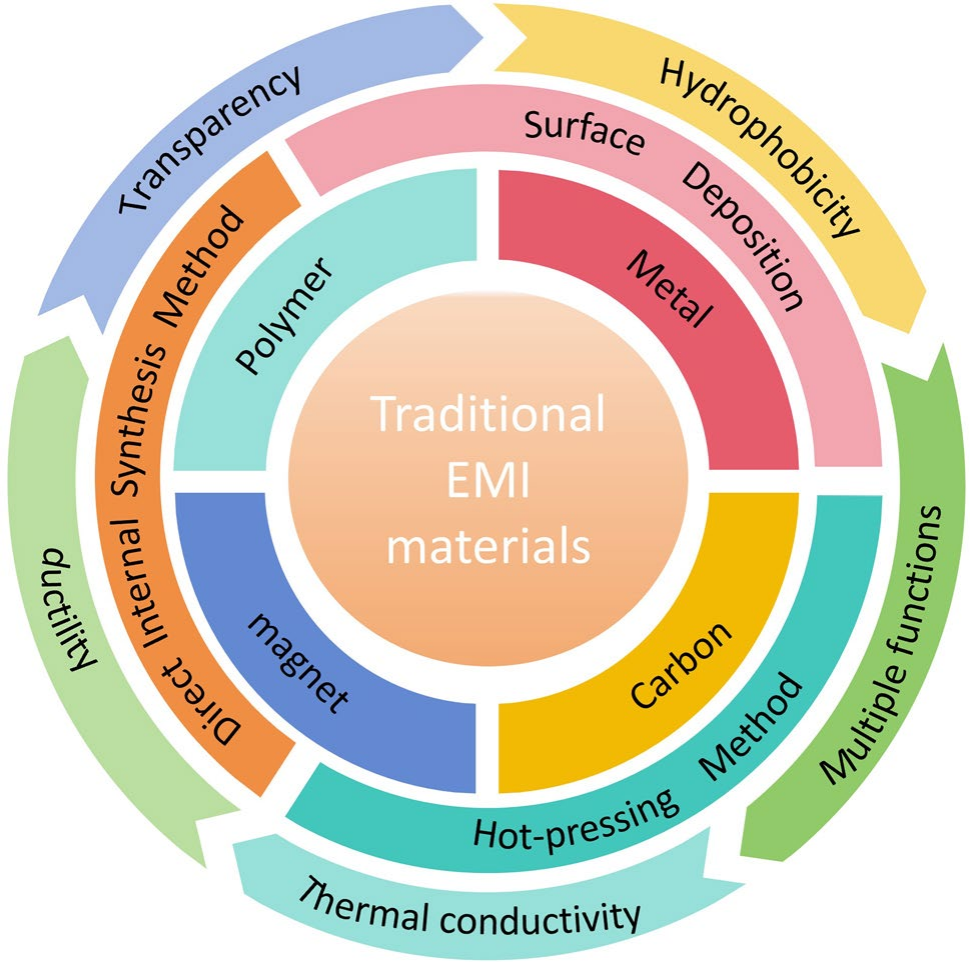
Traditional EMI shielding materials, preparation methods and properties

In the past, studies of electromagnetic shielding materials focused on metal oxides [28–30], metals [25, 31], carbon-based materials [32–35], metal carbide [36], sulfide [37], magnetic materials, and polymer shielding materials (Fig. 2) [38–43]. Among these, metals (e.g., Fe, Ag, Ni, Cu, and Al) and their compounds have been extensively studied for their effectiveness in shielding electromagnetic and electrostatic fields [44–46]. It was found that transition metal sulfides exhibit strong electrochemical activity, higher specific capacitance, and enhanced conductivity [47, 48]. However, challenges related to the strong electromagnetic waves (EMWs) that has caused secondary interference, depletion of metal resources, high density, susceptibility to corrosion, and processing difficulties have constrained their widespread application [37, 49–51]. Magnetic materials exhibit strong absorption and attenuation properties when exposed to low-frequency electromagnetic radiation [52, 53]. However, their effectiveness diminishes when exposed to high-frequency electromagnetic radiation, and their thickness further restricts their practical application in electromagnetic shielding [54].

Recently, the primary research direction for polymer shielding materials centers around polythiophene (PT), polyurethane (PU), polypyrrole (PPy), polyacetylene (PA), and other polymers with conjugated π-bonds [55–57]. These materials have secured substantial spotlight due to their outstanding performance, which includes high efficiency, lightweight, corrosion resistance, and excellent processing capabilities [58]. For example, Sun et al. used electrostatic assembly and molding to load Ti_3_C_2_ onto the surface of polystyrene particles. Due to the high conductivity of MXene and its efficient conductive network in the polystyrene matrix, the polystyrene/Ti_3_C_2_ composite was constructed with a high conductivity of 1081 S m^−1^ and an electromagnetic interference shielding effectiveness (EMI SE) of 64 dB [59]. However, the preparation process is complex, and the individual materials do not possess exceptional electromagnetic shielding performance. As such, large amount of conductive fillers is usually added, which in turn limits its application in electromagnetic shielding and compromises the mechanical properties of the material [60–62].

On the other hand, carbon-based materials like reduced graphene oxide (RGO), carbon nanofibers (CNF), carbon nanotubes (CNT), and their composite materials [63–68] exhibit excellent electrical conductivity, high dielectric loss, specific surface area, outstanding chemical stability, and large aspect ratio, suggesting their potential use in electromagnetic shielding applications [69–72]. For example, Li et al. successfully constructed a CNT/SiC coaxial three-dimensional porous composite sponge using a low-temperature growth strategy. Its comprehensive performance is excellent, low density, super elasticity, excellent thermal resistivity, EMI of 75.7 dB in the X-band [73]. However, their relatively high production costs, expensive manufacturing equipment, and complex processing methods present challenges in meeting large-scale production requirements [60, 74].

### Application of Biomass Materials in Electromagnetic Shielding Field

**Fig. 3:**
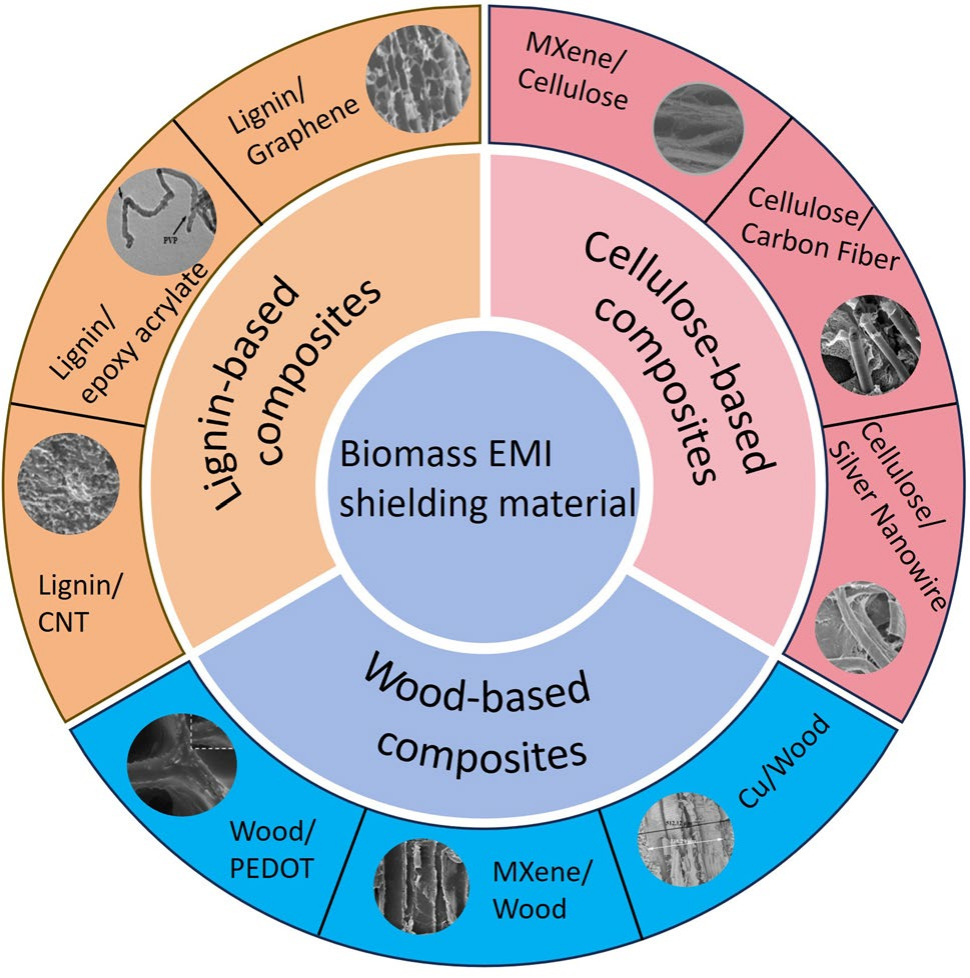
Various types of biomass materials for EMI shielding field, with wood, cellulose, lignin as an example

The call for effective electromagnetic shielding materials has elevated in recent years owing to resource scarcity and growing environmental concerns. Unfortunately, existing traditional materials have struggled to meet practical demand, but problems such as their difficulty in processing, non-degradability, and depletion of raw materials have led to the exploration of alternative options [75–77]. Biomass materials have garnered attention due to their low cost, sustainability, lightweight nature, and porous hierarchical structure, making them a promising alternative to traditional EMI shielding materials [78–81]. Biomass-based multi-function electromagnetic shielding materials not only effectively shield electromagnetic waves, but also have other functions, such as electrical conductivity, significant flame retardancy and antibacterial activity. Compared with traditional materials, biomass materials can adjust their structure through different treatment and processing methods, for example, by optimizing the pore structure, shape, size and distribution to improve the material's absorption loss and multiple reflection attenuation, thereby enhancing its shielding effect. Some common biomass materials used for electromagnetic shielding are wood, bamboo, lignin, and cellulose (Fig. 3). In particular, wood-based composites are known for their excellent electrical conductivity, lightweight and stable structure, and porous nature, making them a strong candidate for EMI shielding.

Wood-based materials such as wood metal composites, wood polymer composites, and wood-derived carbon composites are employed in EMI shielding [82–84]. Additionally, graphene is a demanding option for EMI materials due to its extensive surface area, exceptional electrical conductivity, mechanical flexibility, and other remarkable physical and chemical properties [85]. For instance, Guo et al. prepared Wood/Cu-Fe_3_O_4_@ graphene /Ni composites. The conductivity of the composite was improved by adding Fe_3_O_4_@graphene with high crystallinity and purity. The micro and nano particles are evenly distributed on the surface of the wood, forming a dense coating. The EMI SE of the composite material is 96.79 dB [86]. Furthermore, nanocellulose (i.e., a natural polymer derived from cellulose), which has a high specific surface area and impressive mechanical properties [87, 88], shows promise for application in biomass EMI shielding materials [31, 89, 90]. For example, Han et al. utilized magnetic Ni particles to modify graphene oxide and nano-fibrillated cellulose in order to create EMI shielding films. Their research found that EMI shielding effectiveness (SE) could reach 32.2 dB [91]. Additionally, Zhang et al. utilized bamboo as a renewable biomass material to prepare a bamboo-plastic composite electromagnetic shielding material. By filling high-density polyethylene (HDPE) with nickel-plated bamboo, they achieved an impressive EMI SE of 82 dB [92]. Moreover, cellulose and lignin were highlighted for their wide availability, low cost, and porous nature [93]. Exploration of a wide range of materials including wood-based composites, graphene-enhanced polymers, and bamboo-plastic composites [94], demonstrates the potential for high levels of EMI shielding effectiveness. These innovative approaches address the urgent need for effective EMI shielding and demonstrate the potential of using renewable biomass materials to develop sustainable solutions for mitigating electromagnetic pollution.

Currently, there is a growing concern for the environment and an increased awareness of the importance of developing effective wearable protective materials and EMI shielding materials [95, 96]. For example, Yuan et al. have documented the development of a highly elastic, stretchy polyurethane nanofiber fabric coated with Ti_3_C_2_T_x_, which maintains an EMI SE of over 20 dB and exhibits stable mechanical properties [97]. Cao et al. have also introduced a composite film utilizing CNTs/MXene/CNFs to create wearable yet flexible EMI shielding materials through a sandwiching process [98]. Similarly, Zhao et al. obtained the polyacrylamide/2-hydroxypropyl trimethylammonium chloride chitosan (PAM/HACC) interpermeable network by heat-initiated polymerization through the strong electrostatic interaction and hydrogen bonding between the positively charged group on HACC and the PAM polymer chain. It was used as skeleton in situ polymerization of PPy. It has flexibility, good mechanical strength, and EMI SE up to 40 dB [99]. Biomass materials have delineated a remarkable possibility for EMI shielding due to their rich interface and porous structure. This allows them to achieve EMI SE of over 20 dB. Furthermore, these materials can be tailored to provide resistance to mildew, electrical conductivity, and flame retardancy, making them applicable for use in various extreme environments [68, 100]. Despite their promising characteristics, biomass-based EMI shielding materials have limited reported applications, hence suggesting significant untapped potential [101–103]. Therefore, there is an urgent need to explore and review existing works related to these promising biomass materials for EMI shielding.

This paper comprehensively reviews preparation methods, material structure design, EMI shielding mechanisms, and other relevant aspects of various biomass materials. A detailed summary of recent research on different types of biomass EMI shielding materials was proposed, along with an analysis of the associated challenges, issues, and future trends. The review emphasizes recent advancements and noteworthy accomplishments in applying biomass-based materials in electromagnetic shielding. It is anticipated that this review will significantly influence the development of environmentally friendly, lightweight, and sustainable electromagnetic shielding materials. The content provided would inspire contemporary design for the creation of relevant biomass electromagnetic shielding materials and propose new possibilities for the design of green yet degradable biomass materials with excellent electromagnetic shielding properties that can be used in various industries such as construction, medical treatment, and clothing [104–106].

## Mechanism of Electromagnetic Shielding

**Fig. 4:**
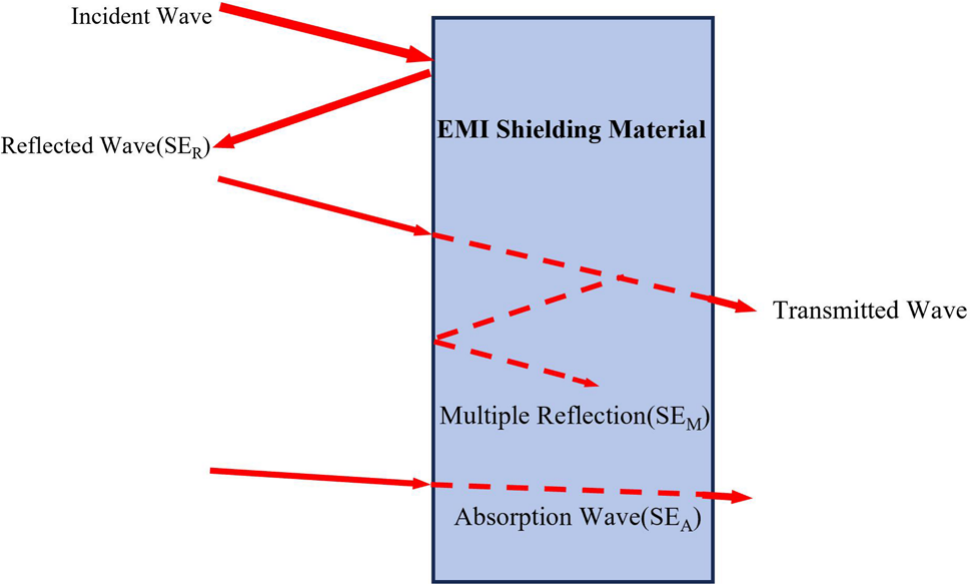
Electromagnetic shielding mechanism diagram of EMI shielding material

When a magnetic field changes, it causes an electric field to change. These fields oscillate vertically in the same direction and create electromagnetic waves (EMW) in the changing field [107, 108]. Unlike other types of wave propagation, EMWs transfer energy efficiently without a medium. The resulting electromagnetic radiation from EMWs has an irreversible impact on the surrounding environment. This effect is known as EMI. EMI shielding involves using specific materials to isolate EMWs and effectively control their transmission in a certain area. The shielding mechanism includes internal multiple reflections, reflection, and absorption (Fig. 4).

### Shielding Effectiveness

EMI SE represents the primary indicator to evaluate the electromagnetic shielding effect of materials. The EMI SE principally relies on the internal multiple reflection loss, absorption loss and reflection loss of electromagnetic shielding material.

EMI SE defines the ratio of the intensity of the incident electromagnetic field to the emitted electromagnetic field and is expressed by Eq. (1) [109–111]:

1 $$ SE = 10\log \left( {\frac{{P_{i} }}{{P_{t} }}} \right) = 20\log \left( {\frac{{H_{i} }}{{H_{t} }}} \right) = 20\log \left( {\frac{{E_{i} }}{{E_{t} }}} \right) $$

where *P*_*t*_ denotes the transmitted EMW power, *P*_*i*_ is the incident EMW power, *H*_i_ represents the incident EMW magnetic field, *H*_t_ refers to the transmitted EMW magnetic field, *E*_t_ denotes the transmitted EMW electric field and *E*_i_ is the incident EMW electric field.

According to Serkunov's theory, the EMI SE can be defined in Eq. (2) [112]:

2 $${SE}_{T}={SE}_{R}+{SE}_{A}+{SE}_{M}$$

where *SE*_*T*_ is the total EMI shielding effectiveness, *SE*_*R*_ denotes the surface reflection, *SE*_*A*_ represents the internal absorption and *SE*_*M*_ refers to the multiple internal reflection.

Surface reflection (*SE*_*R*_) is influenced by a mismatch among the intrinsic impedance of the EMI shielding material and the free-space impedance. *SE*_*R*_ can be calculated by Eq. (3) [113–115]:

3 $$ SE_{R} = 168.2 + 10\log \left( {\frac{{\sigma_{rel} }}{{f\mu _{rel} }}} \right) $$

where *f* is the frequency of the incident battery wave, *σ*_*rel*_ denotes the relative conductivity, and *μ*_*rel*_ refers to the relative permeability.

Multiple internal reflection (*SE*_*M*_) is caused by macroscopic multiple reflections within the two shielding layer interfaces. *SE*_*M*_ can be obtained via Eq. (4) as follows [116–119]:

4 $$ SE_{M} = 20\log \left( {1 - 10^{{ - \frac{{SE_{A} }}{10}}} } \right) $$

When *SE*_T_ > 15 dB, *SE*_M_ can be ignored [120].

Internal absorption (*SE*_*A*_) represents the attenuation of electromagnetic energy influenced by magnetic loss and dielectric loss in the EMI shield. The calculation is demonstrated in Eq. (5) as follows [121, 122]:

5 $${SE}_{A}=131.43t\sqrt{f{\sigma }_{rel}{\mu }_{rel}}$$

where *t* denotes the thickness of the EMI shield.

### Effect of Porous Structure of Biomass Materials on EMI Shielding Properties

**Fig. 5:**
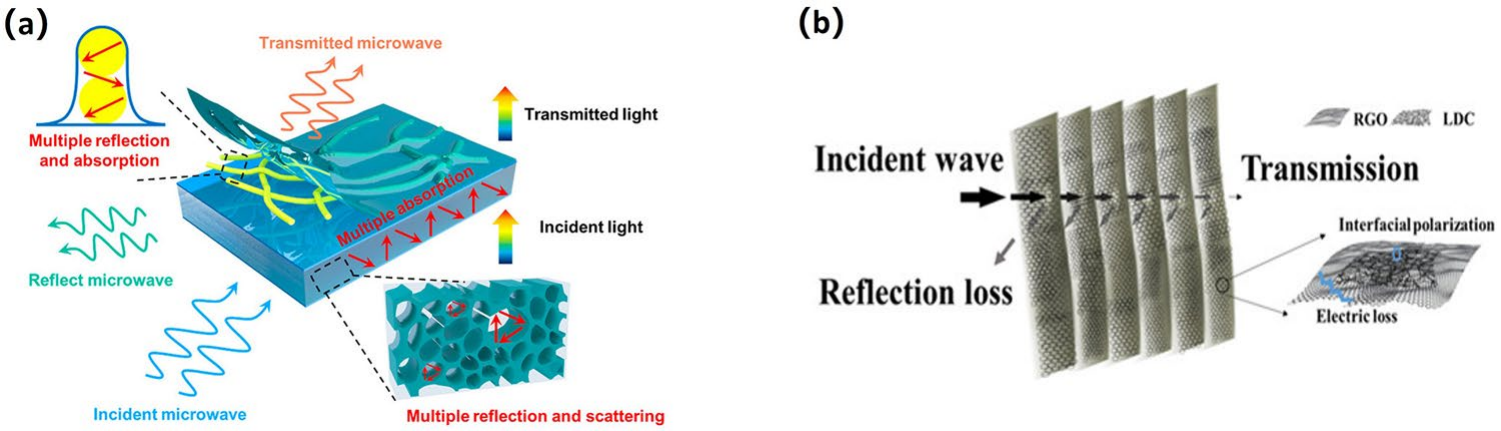
**a** Diagram of electromagnetic shielding mechanism of AgNW@MXene/Wood [125]. **b** Microwave attenuation mechanisms of Graphene/lignin-derived carbon [126]

Figure 5 demonstrates the electromagnetic shielding mechanism of biomass materials. When an electromagnetic wave comes to the shielding material surface, a portion of the wave is reflected due to the mismatch between the impedance of the travelling medium its inherent impedance, hence reducing the energy that passes through the interface. The remaining energy is absorbed by the shielding material and converted into heat, this further reducing the EMW. The remaining electromagnetic wave is gradually attenuated through multiple reflections inside the shield, with only a small amount passing through the material [107, 123, 124]. The higher the conductivity and magnetic permeability of the shielding material, the better the shielding effect. In addition, high-frequency electromagnetic waves have a skin effect in conductive media, resulting in energy loss and reduced field amplitude, making it easier to shield. Overall, the microstructure of biomass materials can effectively shield EMI through the reconfiguration and scattering of EMWs. Adding conductive fillers to biomass materials, such as carbon fiber and graphene, will form a conductive mesh structure. Stacking different polymer composite layers can optimize impedance matching, improve absorption losses and multiple reflection attenuation, and thus enhance the shielding effect. Biomass materials such as crop straw can be converted into conductive carbon after high temperature carbonization, which can improve the EMI SE of composite materials. The porous structure of biomass materials combined with various reflection and absorption processes would block the majority of the electromagnetic waves passing through the material.

**Fig. 6:**
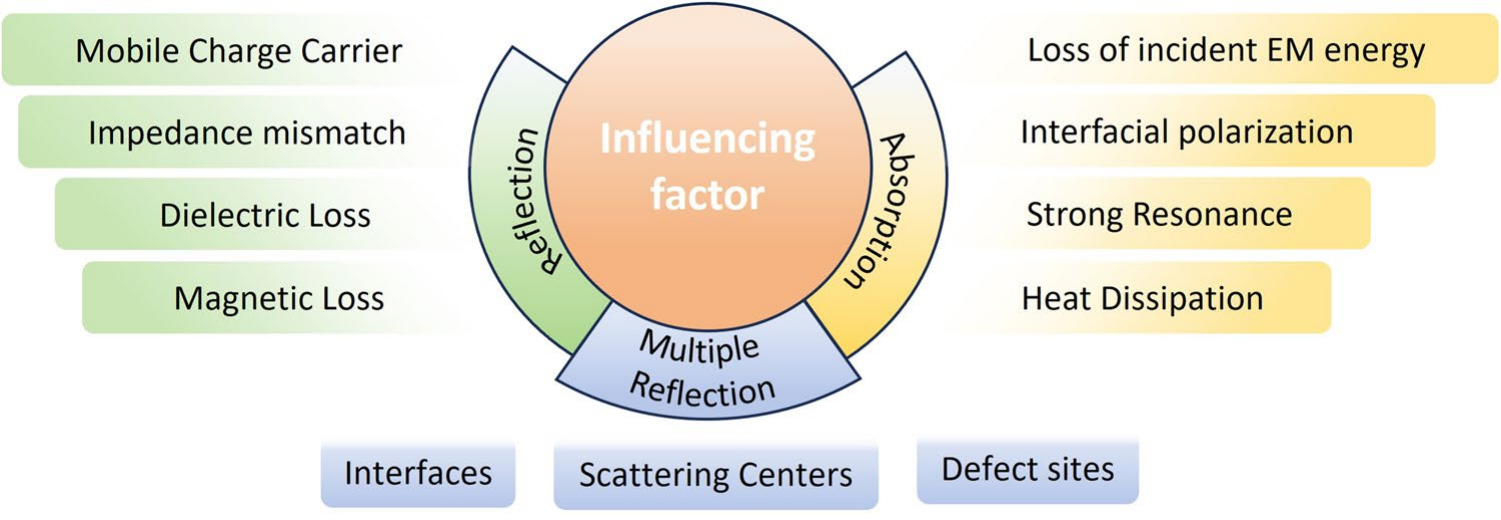
Factors affecting reflection, absorption and multiple reflection in EMI shielding

Figure 6 illustrates the factors that affect reflection, absorption, and multiple reflections in EMI shielding. By adding conductive fillers, stacking different composite layers and carbonization treatment, the biomass material has high magnetic conductivity. The composite material can selectively shield EMW in EMI shielding field. The EMI SE of hybrid polymer composites is influenced by several properties and factors, including the magnetic and electrical properties of fillers, fibers, and polymer substrates, as well as the manufacturing methods and composite structure. Composites typically have a combined shielding mechanism of reflection and absorption at the interface, followed by absorption. As electromagnetic waves propagate through the material, they scatter at scattering centers, interfaces, or defects, leading to electromagnetic radiation attenuation [127].

The EMWs inside the material decay due to a variety of mechanisms, mainly including dielectric and magnetic losses, which convert electromagnetic energy into heat. Dielectric loss is when EMWs pass through biomass material, the atoms or molecules in the medium are vibrated by the electric field, reducing EMW propagation. Magnetic loss is the energy loss caused by the change in magnetic field energy during EMW propagation, which is converted into heat energy, thereby improving EMW absorption. In experiments, multilayer shielding structures are usually designed to achieve impedance matching. Interfacial polarization is the polarization charge induced at the material interface due to uneven charge distribution, which will produce a dissipative effect under an alternating electric field. Moreover, Polarization loss is the energy loss caused by dielectric polarization under electric field action [128]. In experiments, multilayer shielding structures are usually designed to achieve impedance matching. Improved impedance matching can reduce the EMW reflection on the material surface so that the EMWs enter the material and are absorbed [129]. Through the application of these mechanisms, we can better prepare biomass composites with high significant EMI SE. For example, the overall absorption effect can be enhanced by designing multi-layer structures that take advantage of the impedance and absorption characteristics of different materials.

## Preparation and Characterization

There is a significant amount of literature on the EMI shielding properties of biomass materials. A summary of various biomass materials used for electromagnetic shielding is available. The following section will describe the preparation and properties of biomass materials for EMI shielding.

### Wood and Its Derivatives

Wood represents an environmentally friendly yet biodegradable material with a large specific surface area and rich layered porous structure [130, 131]. The presence of these pores improves the impedance matching performance, improves the absorption efficiency of EMWs and facilitates multiple EMWs reflection in the pores. Besides, the wood surface also consists of abundant active hydroxyl groups, which provides an ideal environment for binding of inorganic particles [132–134].

#### MXene Compounding

MXene is a class of two-dimensional nanoscale transition metal carbides whose excellent metal conductivity, chemical stability, a high density of electron states and adjustable surface functional groups make them suitable for EMI shielding [135–138]. However, the poor mechanical properties of MXene materials lead to oxidation and degradation in humid environments, which in turn limits their effectiveness in applications requiring electromagnetic shielding [139–141].

**Fig. 7:**
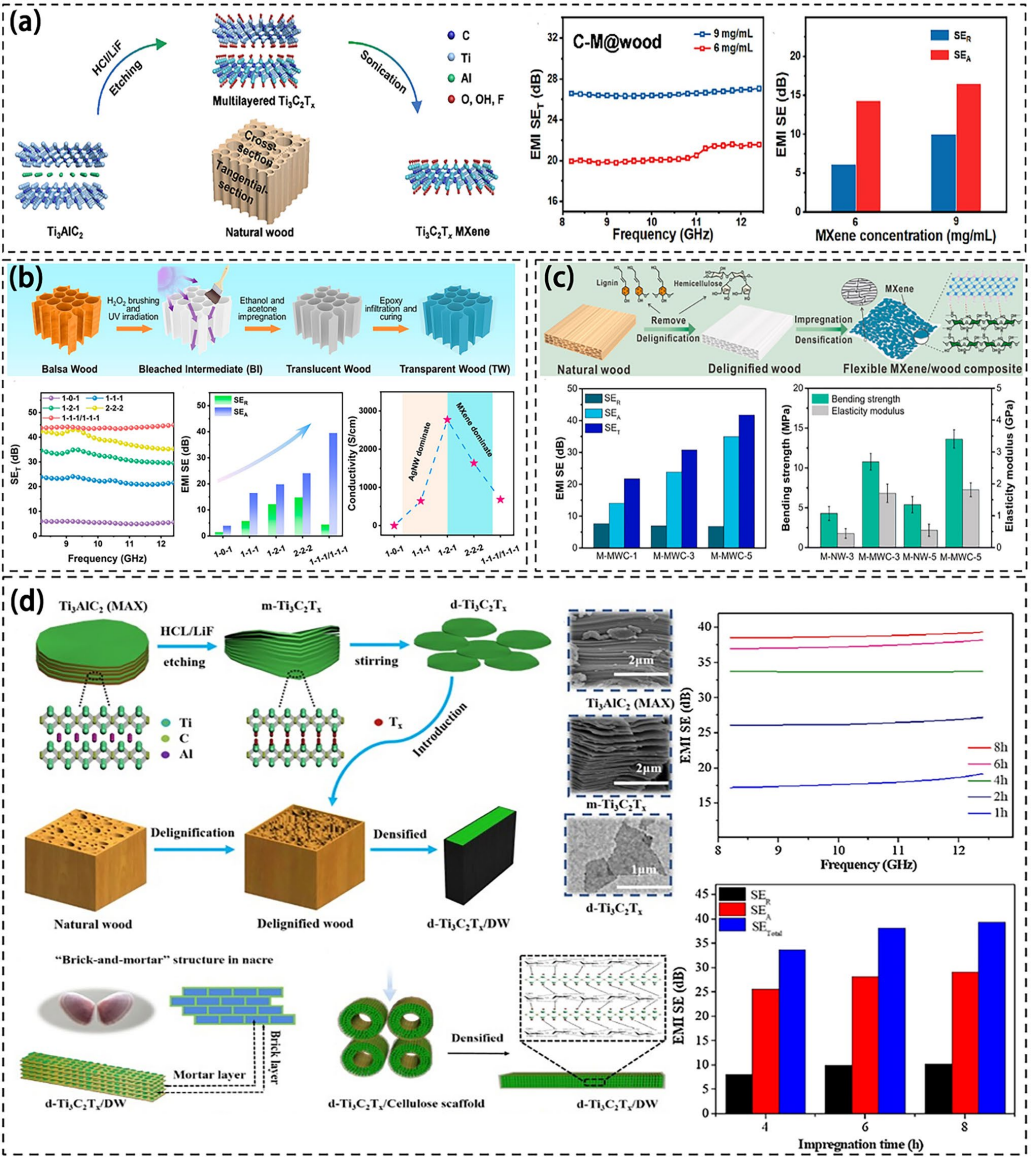
**a** Preparation of highly anisotropic MXene@Wood composites and EMI shielding properties of cross sections [142]. **b** Illustration of the preparation process of WA-M/wood [125]. **c** Schematic diagram illustrating the fabrication for Flexible MXene/wood composite and EMI shielding performance of composite [145]. **d** Preparation and characterization of the d-Ti_3_C_2_T_x_/DW and EMI shielding performance of the d-Ti_3_C_2_T_x_/DW [147]

Figure 7 depicts the fabrication process of MXene/wood composites and their electromagnetic shielding properties. Wei et al. applied MXene coating on both the tangential-section and cross-section of wood. They discovered that the impedance matching of the tangential-section was higher than the cross-section. This disparity was due to the unique pore structure of wood and the presence of free radicals (Fig. 7a) [142]. In addition, Wei et al. prepared MXene/wood composites by applying MXene on the wood surface and then coating it with self-crosslinking polyurethane-modified waterborne acrylic resin to achieve waterproof performance. Remarkably, the EMI SE of the composite reached 31.1 dB [143]. The study broadens the application prospects of MXene/wood composites and solves the problem of oxidization and degradation in oxidization and degradation in humid environments. The spraying method is an expandable preparation technique applied to poplar, pine, and lychee wood. The EMI shielding requirements can be achieved commercially after 3–5 coats. Similarly, Cheng et al. employed UV-assisted chemical and mechanical spraying techniques to apply a coating of silver nanowires (AgNW) and MXene onto transparent wood. This process resulted in a sandwich composite material. They introduced structural shielding through a multilayer stacking method, leading to an EMI SE of 44 dB (Fig. 7b) [125]. In this case, the AgNW was sprayed on the MXene surface to enhance the electrical conductivity, and the ordered microtubule channel array of transparent wood induced multiple reflections of EMWs to enhance the EMI shielding performance [144].

Similarly, Jiang et al. prepared MXene/wood composites by immersing wood veneer boards with lignin removed by acid treatment into MXene suspension, which were hot-pressed into alternating multilayer structures, resulting in significant EMI shielding capability with an EMI SE of 32.7 dB (Fig. 7c) [145]. It is worth noting that as the MXene load increases, the EMI SE gradually increases. The EMI SE reaches its maximum when the load is 6.7 mg cm^−3^. The experimental densification process enhances the flame retardancy and mechanical stability of the material and produces a dense conductive network that contributes to the absorption and reflection of EMW [146]. The surface of MXene/wood is noticeably smoother than natural wood, with MXene covering the narrow cracks. This MXene layer expedites electron transfer among neighboring MXene sheets, effectively creating a continuous conductive path.

Wang et al. developed a simple top-down method to transfer d-Ti_3_C_2_T_x_ nanosheets onto cellulose scaffolds using vacuum pressure-assisted impregnation to make densely layered d-Ti_3_C_2_T_x_/cellulose scaffolds, which resulted in excellent mechanical and EMI shielding properties with an EMI SE of 39.3 dB [147]. The microstructure of d-Ti_3_C_2_T_x_/wood composites prepared in this experiment has an ordered laminar structure, which ensures the mechanical properties of the material and the reflection and absorption of battery waves. The pearly layered microstructure allows the remaining electromagnetic waves to combine with the high electron density MXene layer and multiple internal reflections in the wood, allowing energy dissipation and absorption of EMWs [147, 148]. With the increase of impregnate time, the content of* d*-Ti_3_C_2_T_x_ was escalated, and the corresponding EMI SE was also enhanced. The maximum shielding efficiencies of the d-Ti_3_C_2_T_x_/wood composites with diverse impregnate periods were higher than 99.95%.

MXene/wood composite methods are divided into two categories: impregnation method and spraying method. The primary objective of adding MXene to biomass materials is to create electric dipoles and interfacial polarization, thus affecting the propagation of EMWs. The EMI SE of the composites obtained is more than 30 dB, which meets the EMI shielding requirements of common industrial electronic instruments. The use of MXene composite offers a novel approach to creating electromagnetic shielding materials from biomass and opens up new possibilities for preparation methods. Research has demonstrated that MXene/wood composites can be tailored to meet specific property requirements, including mechanical strength, flame retardancy, flexibility, and transparency [149–151]. This advancement significantly broadens the potential applications of MXene/wood composites in EMI shielding, making them appropriate for application in extreme environments and presenting exciting opportunities for the future [152, 153].

The integration of MXene with wood is showing great promise for EMI shielding materials [154]. While MXene possesses remarkable conductivity and chemical stability, its poor mechanical properties in humid conditions pose a challenge. Various methods such as impregnation and spraying techniques, have been explored to address this limitation. The resulting MXene/wood composites have demonstrated significant EMI shielding capabilities exceeding 30 dB. These composites possess ordered microstructures that improve mechanical stability and EMI SE through electromagnetic wave reflection, absorption, and multiple internal reflections. Customizing MXene/wood composites may offer flexibility in meeting specific property requirements and expanding their potential applications across various industries [155].

#### Metal Compounding

**Fig. 8:**
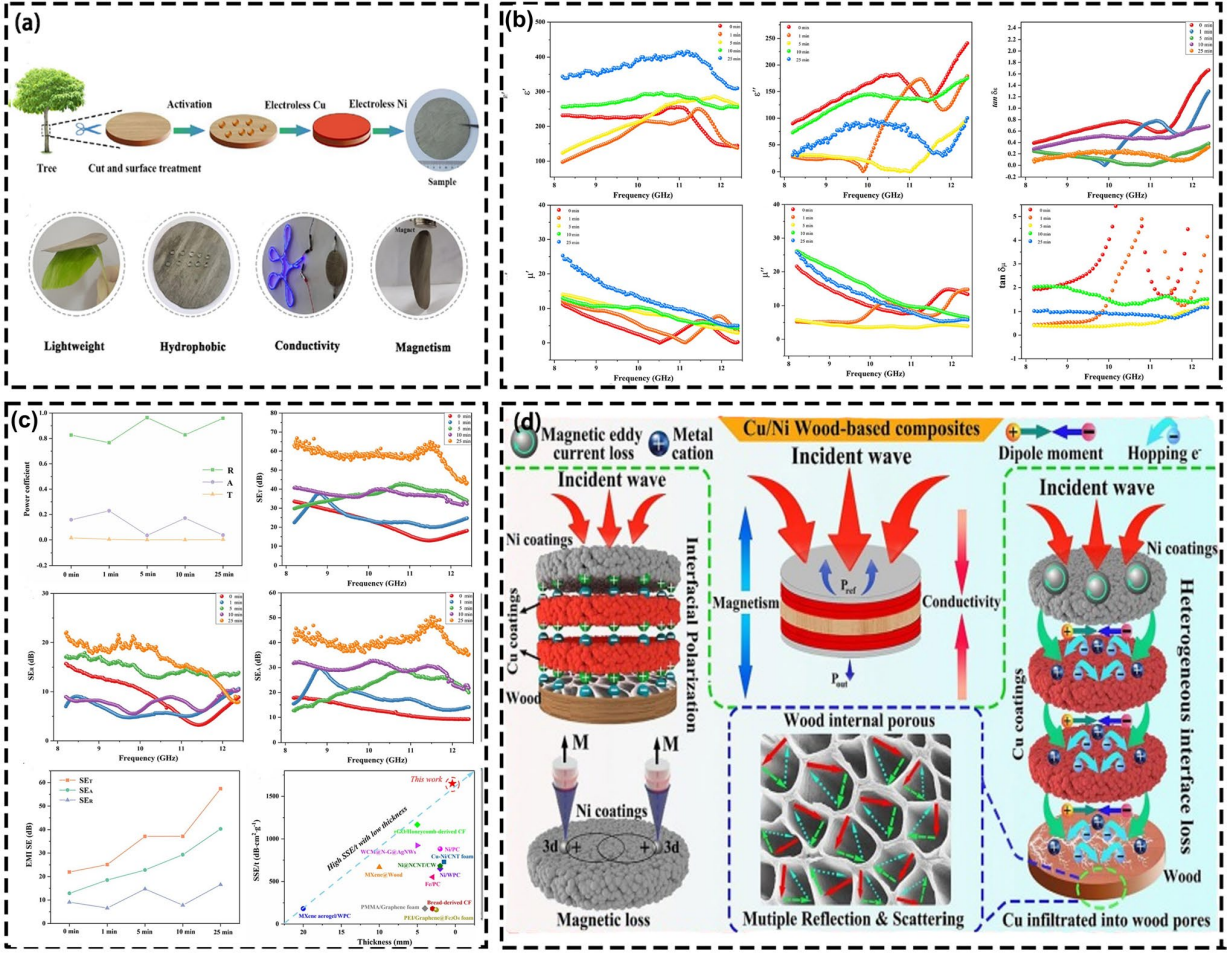
Sandwich-structured Cu-Ni wood-based composites. **a** Preparation of Cu-Ni wood-based composites; **b** Electromagnetic parameters, magnetic loss tangent and dielectric loss tangent of each sample; **c** EMI shielding properties of sandwich-structured Cu-Ni wood-based composites; **d** Schematic illustration of absorption mechanism of Cu-Ni wood-based composites [164]

Figure 8a–d shows the wood/metal composite preparation process and the EMI SE under different conditions. Pan et al. developed a composite material with highly effective EMI shielding properties by creating a sandwich structure on wood with a nickel-plated surface. The material demonstrated an impressive absorption efficiency of 94.1 dB for EMI shielding which could be attributed to the synergistic impact of surface absorption loss and interfacial polarization loss [156]. When the coating is applied, the composite surface becomes smoother as it tightly integrates the Ni layer with the wood, resulting in a dense composite coating. This coating not only creates an effective conductive network but also enhances the hydrophobic properties of the composite material. Based on the loss mechanism, the Ni/wood composites leverage interfacial polarization loss, conductivity loss, and magnetic loss to absorb incident EMW [46]. However, a relatively large Ni layer thickness is required for better electromagnetic shielding properties. Therefore, it affects the degree of coating uniformity and manufacturing cost.

In addition, Pan et al. used electroless Cu plating to deposit Cu particles on the surface of poplar pasteboards to prepare laminated laminar composites. These composites exhibit absorption-based and reflection-based shielding mechanisms, providing an EMI SE of 96 dB and good hydrophobicity [157]. The multi-interfacial polarization within Cu and wood and the anisotropic internal porous structure of the wood matrix gives the prepared composites excellent EMI shielding properties [158]. As the duration of electroless Cu plating increases, the wood surface texture becomes smooth and the uneven pores are covered by metal particles, creating a consistent metal layer that forms a reliable conductive network. The presence of numerous interfaces between adjacent conductive networks such as air-Cu, Cu-Cu, and Cu-wood, leads to the absorption of incident EMW through multiple reflections [50, 159]. Additionally, the difference in dielectric constants results in the accumulation of free charge at the non-uniform interface boundary among metallic copper and wood, causing interfacial polarization and the generation of macroscopic dipole moments and Debye relaxation. This ultimately leads to the attenuation of EMW energy [160, 161].

Guo et al. conducted electroless Cu-Ni plating on wood surfaces. Firstly, Cu was deposited onto the wood, resulting in some Cu particles in the activation holes and on the wood surface. Subsequently, a Ni layer was applied to the Cu layer through electroless Ni plating, followed by the deposition of electroless Cu plating on the Ni layer. This process led to the creation of Cu-Ni multilayered composites with an exceptional EMI SE of 93.8 dB. These composites exhibited impressive EMI shielding properties, favorable surface roughness, hydrophobicity, and electrical conductivity [162]. The fine Cu particles can fill the porous structure and surface defects of the wood, promoting the formation of a uniform metal layer on the wood surface. At the same time, the uniform Cu layer provides an autocatalytic substrate for the Ni particles deposition. The Cu and Ni layers on the wood act synergistically for electromagnetic shielding due to the conductive networks and the specific interfacial polarization mechanism of the composite coating and promote the absorption of incident EMW via the polarization of the electric field [163]. Furthermore, Dai et al. prepared a Cu-Ni wood sandwich structure composite with an EMI SE of 57.4 dB by electroless copper-nickel plating. The composite formed a three-dimensional electromagnetic network and possessed an ideal microchannel structure with the copper-nickel coating (Fig. 8) [164]. It was found that the Ni and Cu nanoparticles within the composites form a network that facilitates electron movement, thus decreasing resistivity. The multiple non-uniform interfaces within the Cu, Ni, and wood and Ni layers and air also contribute to interfacial polarization [46].

Electroless plating is the primary method for laminating metal particles with wood, leveraging the abundant pores of wood to infuse metal particles and create a uniform metal layer on the surface. Copper (Cu) and nickel (Ni) are commonly used metal particles. The integration of metal particles into biomass materials significantly enhances magnetic loss and EMW absorption capabilities while also improving electrical conductivity, impedance matching, and interface polarization within the composite [165]. The combination of wood-metal complexes with their porous wood structure and interfacial polarization facilitated by the metal layer exceeds commercial EMI shielding requirements with over 90 dB. This exceptional shielding effectiveness not only meets but surpasses the strict demands of sensitive instruments for EMI shielding, representing a significant advancement in the field.

#### Polymer Compounding

**Fig. 9:**
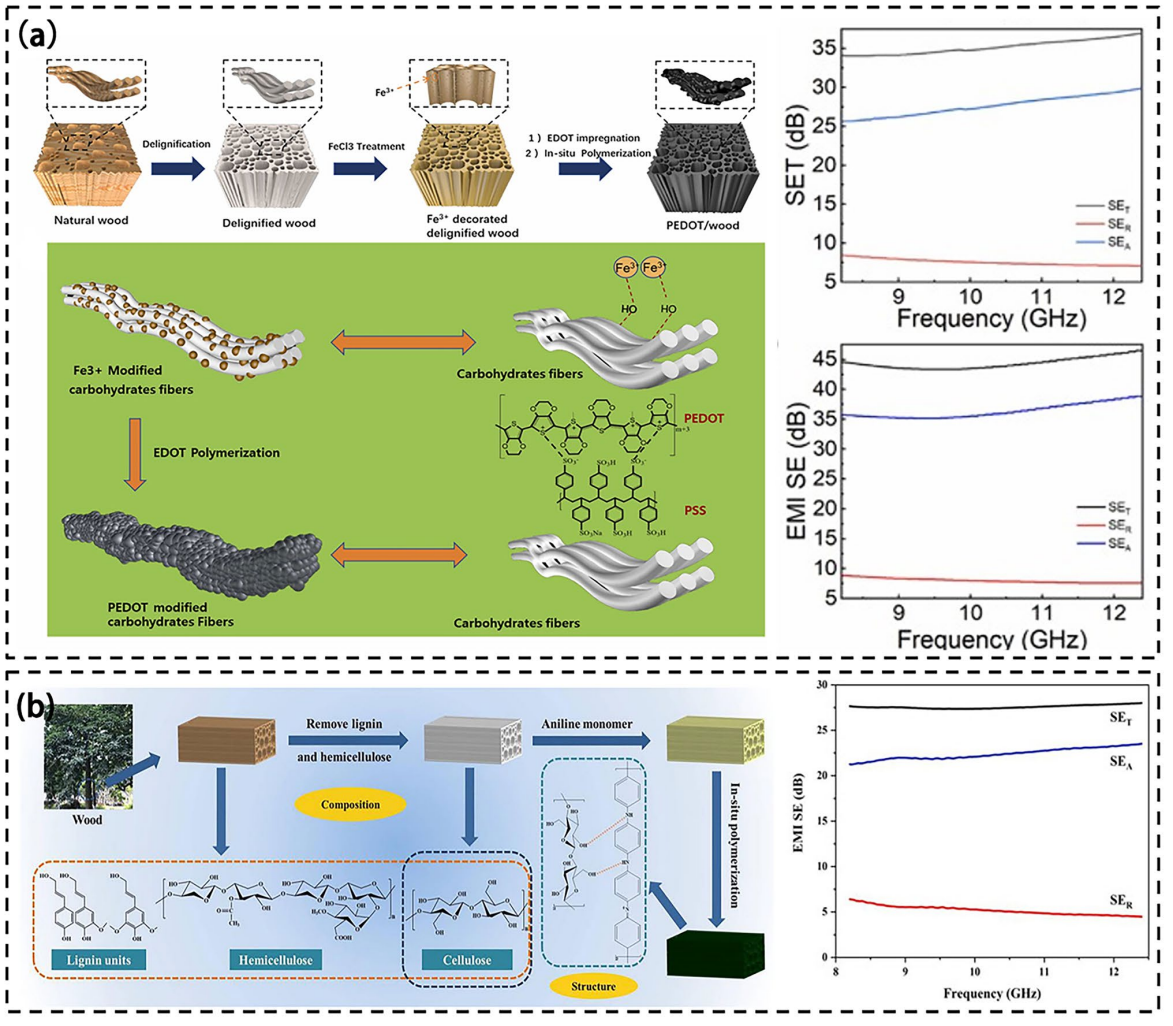
**a** Scheme for preparing PEDOT/wood and EMI shielding performance of composite [167]; **b** Preparation process of PANI-WA aerogel and wood and EMI shielding performance of composite [168]

Figure 9 shows the polymer/wood composite preparation process and their respective EMI SE. Karteri et al. utilized camphor pine wood chips, polyethylene (PE) and graphene nanoparticles to make microspheres through a twin-screw extruder and thermally pressed them to produce wood-polyvinyl chloride/graphene nanoflake nanocomposites with EMI SE of more than 25 dB [166]. This study demonstrates that a higher graphene content in the composite material reduces EMW penetration and enhances shielding efficiency. Specifically, the composite containing 9 wt% graphene exhibits superior EMI shielding performance, with a higher EMW absorption than reflection. This indicates that the material functions as an absorption-based EMI shielding material. In addition, Chen et al. used an in-situ polymerization method to eliminate lignin from wood by chlorination. Then, the monomers were coated on the carbohydrate framework of the delignified wood surface for in-situ polymerization to produce a polythiophene (PT)/wood composite with an EMI SE of 46 dB (Fig. 9a) [167]. The conductive network formed by PT in wood constitutes a continuous current pathway and the conductivity of the composite increases with increasing the mass fraction of thiophene. The shielding effect of its composites is mainly absorption, accounting for 80% of the total shielding effectiveness [158]. Additionally, the material has significantly enhanced mechanical properties, including a compressive strength of 50.9 MPa and a tensile strength of 67.8 MPa.

Additionally, Chen et al. have formed a non-carbonized nanostructured polyaniline (PANI)/wood composite with a 2–3 mm thickness, achieving an impressive EMI SE of 27.63 dB (Fig. 9b) [168]. PANI has an exclusive doping/de-doping and REDOX chemical structure that allows it to transition between insulating and conducting states and attach or detach various anionic groups [169]. This unique property can be harnessed to make wood electrically conductive. Delignified wood was coated with polypyrrole (PPy) via an in situ chemical vapor deposition as reported by Gan et al. The resulting product exhibited a tensile strength of 20.18 MPa and an EMI SE of 21–28 dB. However, the prepared composite has a low mechanical strength and a large thickness, which greatly limits its applicability.

Currently, the predominant method for fabricating wood-plastic electromagnetic shielding composites is in-situ polymerization. The resulting composites rely on electromagnetic wave absorption to achieve effective electromagnetic shielding performance. This is achieved by adjusting the dielectric properties of the polymer to ensure favorable impedance matching with air. Furthermore, the incorporation of conductive fillers facilitates the conductive network formation, thereby enhancing the EMI shielding performance of the material. This approach allows efficient reflection and absorption of EMW, consequently reducing their penetration. However, challenges like low mechanical strength and substantial thickness impose limitations on its practical application.

#### Modified Adhesive

**Fig. 10:**
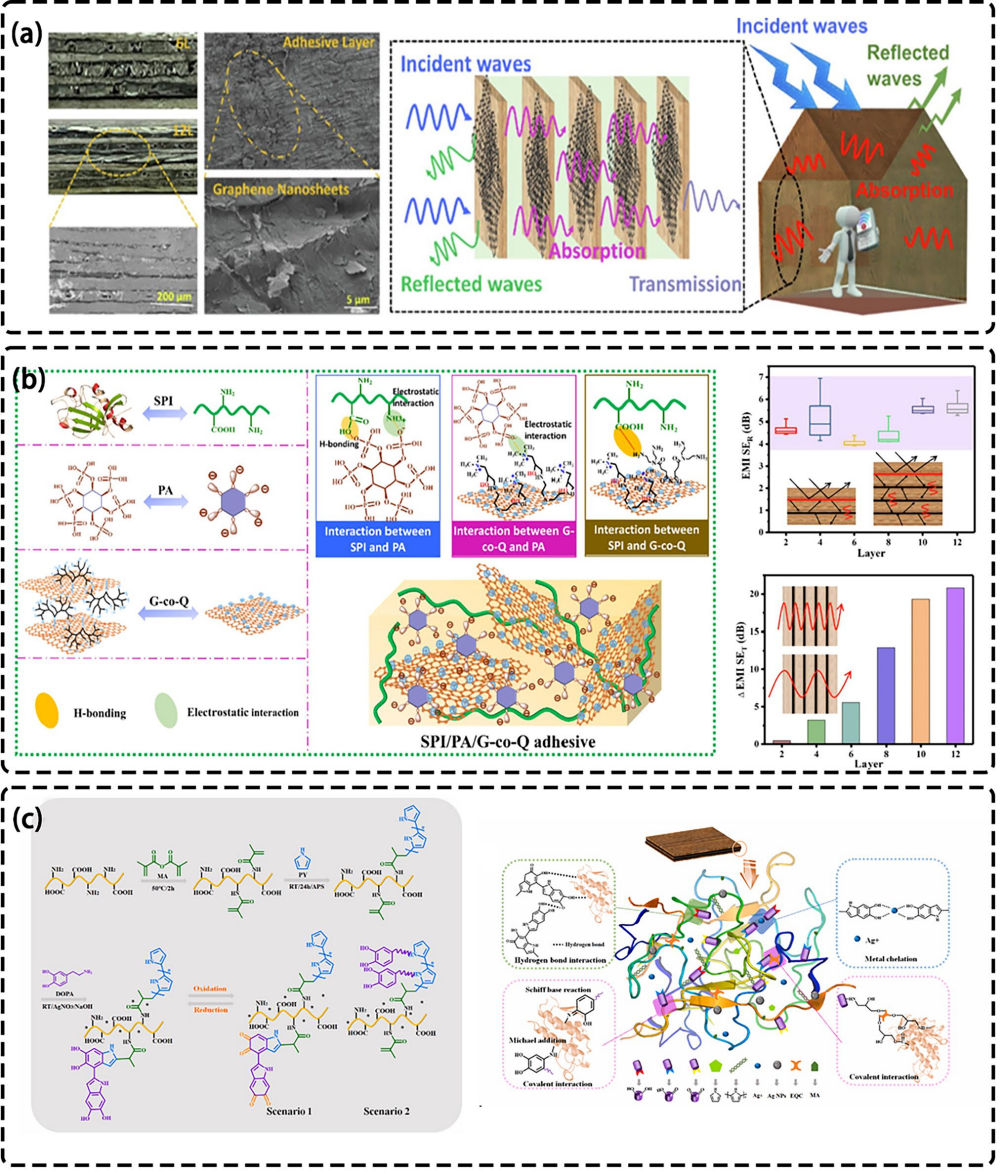
**a** Principle of plywood for electromagnetic shielding [171]. **b** Preparation of organic–inorganic hybrid structures in protein adhesives and EMI shielding properties [171]. **c** Schematic diagram of preparation of strong conductive soybean protein adhesive [172]

Figure 10a shows the principle that plywood can be used for electromagnetic shielding. Ma et al. found that by constructing microporous structure and isolation structure simultaneously, the material can have excellent EMI shielding performance based on absorption [170]. Xu et al. combined antibacterial agent quaternary ammonium salted hyperbranched polyamide (QHBPA) with graphene nanosheets (GNSs) to obtain G-co-Q hybrid [171]. The organic and inorganic hybrid plywood adhesive was prepared by electrostatic interaction and hydrogen bonding with soybean protein isolate (SPI) and phytic acid (PA). It not only shows the electromagnetic shielding performance of 43 dB, but also has good flame retardancy and mold resistance. A conductive layer isolated from each other is formed in the plywood through the adhesive, and the thickness of the conductive layer can be controlled by adjusting the thickness of the veneer to achieve better EMI SE. Among them, GNS is the main reason for its EMI shielding performance. Adjusting the concentration of G-co-Q hybrid ensures the mechanical strength and electromagnetic shielding performance of the plywood, among which, the adhesive containing 7.5% concentration of G-co-Q hybrid has the best performance (Fig. 10b). Similarly, Zhang et al. used dichloromethane, methacrylate anhydride (MA) to modify SPI, and grafted pyrrole (PY) and dopamine hydrochloride (DOPA) on SPI [172]. Ag NPs is generated locally in the adhesive and combined with self-synthesized biological crosslinkers to form hybrid adhesives. Among them, the addition of conductive polymer is the main reason for electromagnetic shielding, when adding 20 wt% PY adhesive, its conductivity can reach 7.09 S cm^−1^, SET is 24.05 dB (Fig. 10c).

There is little research on adhesives that can be used in EMI shielding field. It is mainly the addition of highly conductive polymers to improve the conductivity, so as to meet the commercial requirements of EMI shielding. At present, further exploration of adhesives is still needed to provide better help for plywood in the field of EMI shielding.

### Cellulose and Its Derivatives

Cellulose represents the most widely distributed and the largest reserves of natural polymer materials [173–176]. Cellulose has the advantages of being easily functionalized [177–182], exceptional biocompatibility, environmental friendliness, renewability, and biodegradability [183–185]. It can be easily processed in aqueous solutions, making it a highly versatile and sustainable material with broad applications in various industries [186–192].

**Fig. 11:**
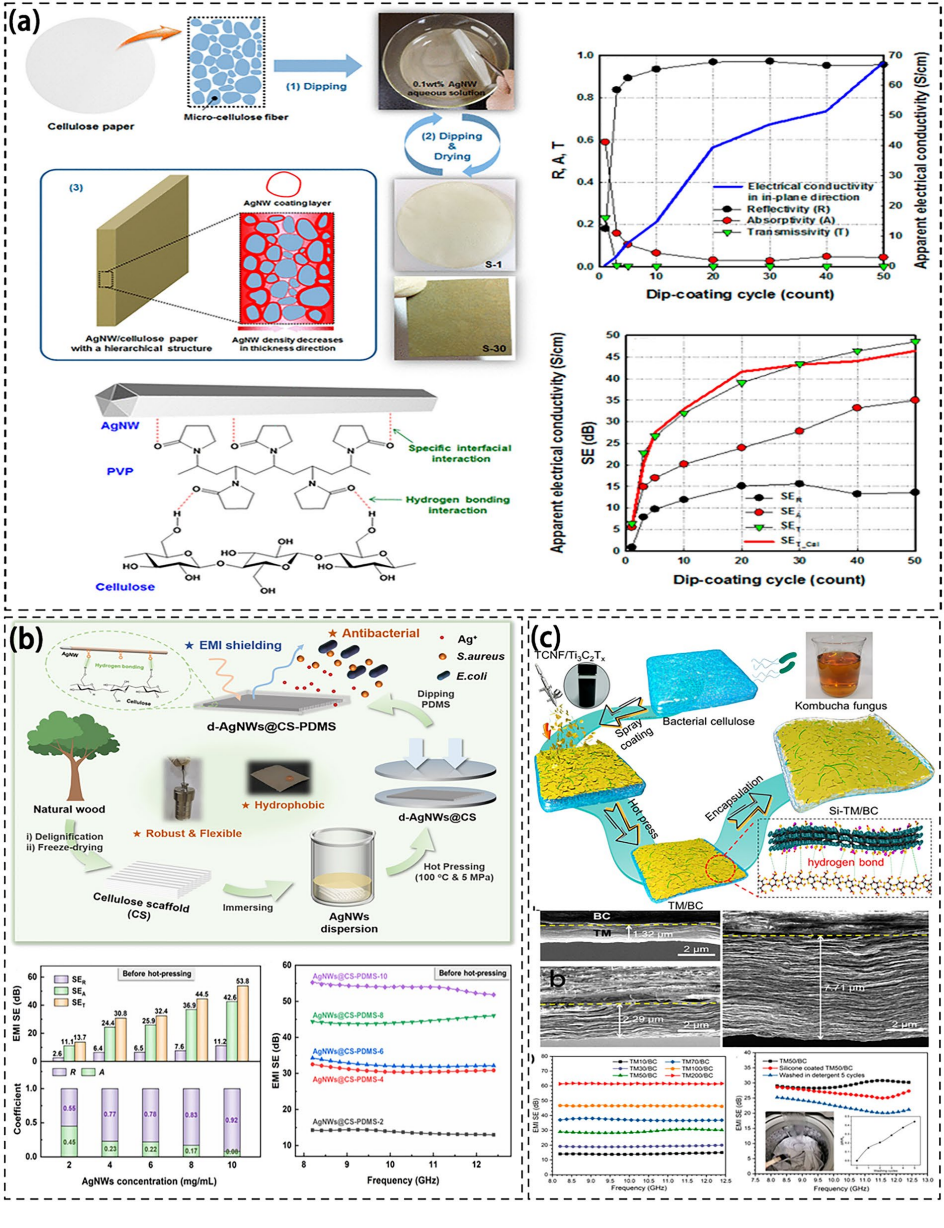
**a** Schematic illustration of the creation process of the composite foams, the foam structure, and EMI shielding performance of composite [194];** b** Preparation and EMI shielding properties of silver nanowire/aligned cellulose scaffold composite [198]; **c** Synthesis of Ti_3_C_2_ MXene/nanocellulose composite films [205]

Figure 10 shows the preparation process of cellulose composites and the effects of different concentrations on EMI SE. Li et al. prepared lightweight EMI shielding cellulose foam/carbon fiber composite material with SE of 60 dB by freeze-drying method [193]. The orientation of fibrous packing within the cell wall of bubbles is influenced by the significant tensile flow that occurs during the growth of the bubble. This flow promotes fiber enrichment and alignment, resulting in a tightly packed foam composite. As the volume expands, the distance between adjacent fibers increases. The short carbon fibers are positioned in the cellulose layer between the bubbles while the long carbon fibers can penetrate the bubbles, thus enhancing their electrical conductivity. Moreover, Lee et al. prepared a layered silver nanowire (AgNW) coated cellulose paper with an EMI SE of 48.6 dB via dip plating (Fig. 11a) [194, 195]. The cellulose fibers in AgNW/cellulose paper are randomly and uniformly coated with interconnected AgNWs. The cellulose paper interior contains AgNWs, forming a continuous conductive network [196]. The exceptional electrical conductivity of AgNWs contributes to the conductive network formation within cellulose paper. As a result, the composite material demonstrates excellent electromagnetic shielding capabilities [197]. Zhu et al. impregnated AgNWs into a highly arranged cellulose scaffold to produce a maximum tensile strength of 511.8 MPa, and an EMI SE of 46 dB [198]. The hydrogen bond between cellulose fibres and AgNWs creates a continuous conductive pathway within the CS microchannel. Through hot-pressing densification, nanofiber alignment is improved, and a more tightly packed conductive network is formed. As a result, this increases dielectric and reflection loss, ultimately enhancing EMI shielding performance (Fig. 11b). Similarly, Xu successfully prepared an efficient EMI shielding film by self-polymerization on CNFs through oxidation of dopamine (DA) and chemical deposition of silver nanoparticles (AgNPs) on CNFs by pressure extrusion process. The resulting composite film has a tightly connected conductive network, which significantly improves the overall conductivity of the EMI shielding film and makes its EMI SE reach 93.8 dB [199].

In addition, Cui et al. created a lightweight composite membrane by combining MXene and CNF using freeze-drying and vacuum filtration techniques. The resulting membrane exhibited excellent mechanical properties, high electrical conductivity, and a highly porous structure, with an EMI SE of 53.7 dB [200]. The CNF was linked with MXene nanosheets through hydrogen bonding, creating a continuous conductive network structure and enhancing the mechanical properties of the composite film [201, 202]. The MXene/CNF composite film demonstrated high conductivity and abundant free charges on its surface, which effectively reflected most of EMW on its surface. As EMW entered the film, it interacted with the high-density charge in MXene while passing through its lattice structure, resulting in ohmic loss and a significant reduction in EMW energy. The porous structure of the composite film expedites multiple reflections of EMW, thereby promoting rapid absorption and attenuation of EMW [203, 204]. Similarly, Zhou et al. achieved an EMI SE of 60 dB by coating MXene on BC nanofiber film using repeated spraying and subsequently resulting in a dense layered nanocellulose film (Fig. 11c) [205]. Using chitin and aramid pulp as raw materials, Zhang et al. prepared chitin cross-linked aramid nanofibers (CANFs) by a room temperature synchronous deprotonation-protonation method, and combined with cross-linked chitin to prepare nanocellulose aerogel (CA). Finally, it was soaked in MXene suspension under vacuum to obtain a uniform porous structure. CA-M aerogel with low thermal conductivity (0.01 W m^−1^ K^−1^) and high EMI SE (75 dB) [206].

Overall, cellulose-based materials for electromagnetic shielding are primarily produced using freeze-drying and dipping methods, resulting in composites with impressive electromagnetic shielding performance. The inherent interfacial polarization in cellulose-based materials enhances EMW loss while the addition of conductive fillers helps the charge distribution and enhances dielectric loss. Additionally, the natural porosity and abundance of hydroxyl groups in cellulose give it strong electrical conductivity, facilitating multiple internal reflections of EMW and improving EMI shielding performance [207]. Through hydrogen bonding, these materials can also achieve excellent mechanical properties and a continuous conductive network structure, rendering them appropriate for a wider utilization.

### Lignin and Its Derivatives

Lignin is a green and renewable biomass material known for its degradability, stability, and low costs [208, 209]. Lignin possesses a porous structure and a complex carbon skeleton structure with numerous benzene rings that contribute to its EMI shielding performance [210, 211]. Additionally, its porous surface is abundant with active sites and functional groups such as free hydroxyl and carboxyl [212], hence allowing it to undergo various chemical reactions with other materials [213–216]. These exceptional chemical properties make lignin an encouraging option for EMI shielding applications.

**Fig. 12:**
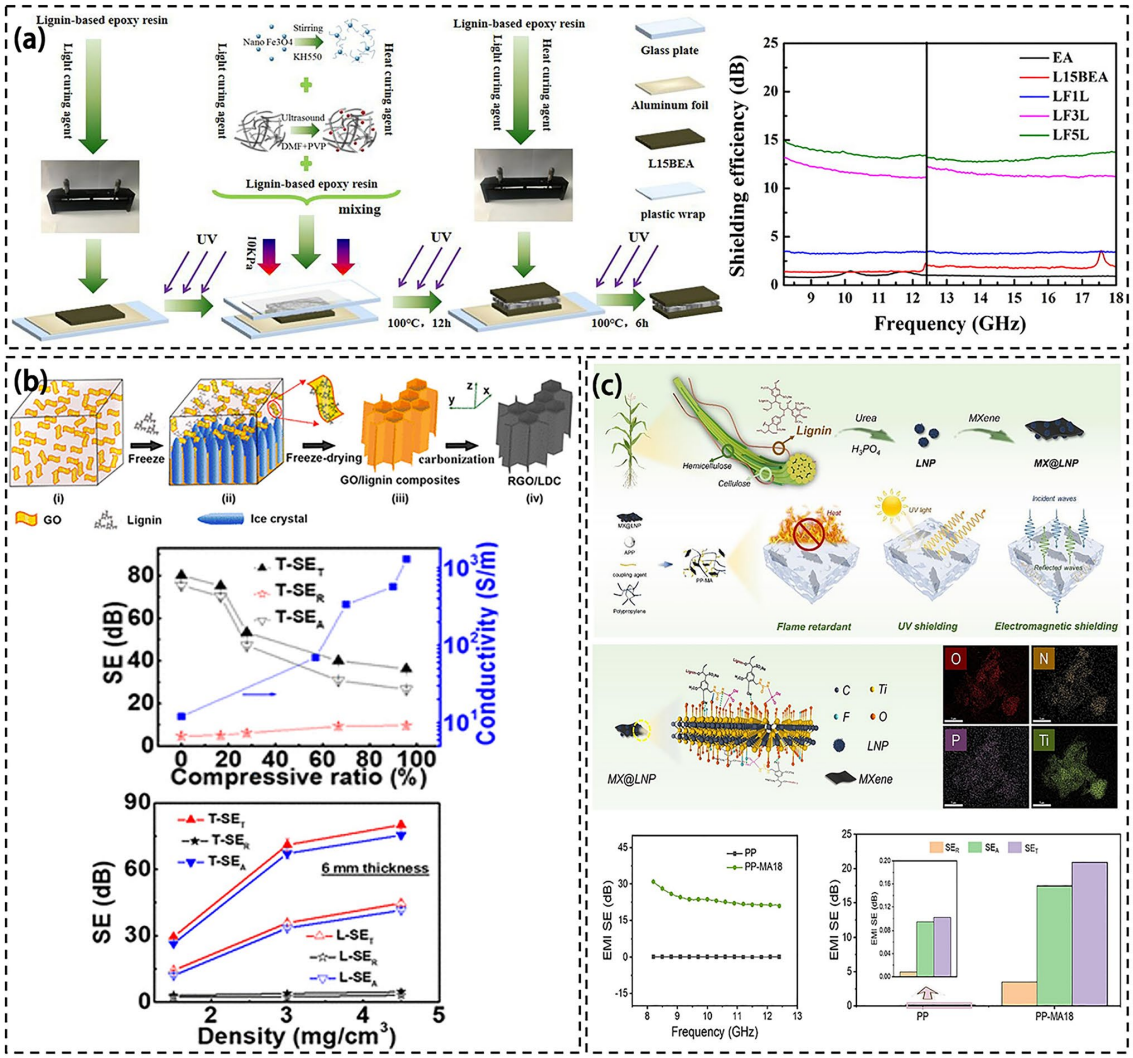
**a** Preparation process of FCLBEA shielding material and EMI shielding performance of composite [220]. **b** Fabrication process of RGO/LDC aerogels and EMI shielding performance of RGO/LDC aerogels [126]. **c** Preparation of multifunctional lignin nanoparticles and their composite structures and electromagnetic shielding properties [223]

Figure 12 illustrates the preparation process of lignin composites and the effect of different concentrations on EMI SE. Zhang et al. used an in-situ insertion method to synthesize lignin-based polyurethanes from graphite, hexamethylene diisocyanate, polyethylene glycol and modified reduced iron powder with lignin. The composite material with 10% iron and graphite content plus 20% lignin content achieved an EMI SE of 22.5 dB [217]. This material exhibits excellent electromagnetic shielding properties, strong mechanical properties, and high thermal stability. It was found that the presence of the phenyl group in lignin creates an opposing magnetic field that shifts and shields the EMW. Additionally, the material can form π bonds with the graphite to enhance the uniform distribution of graphite in the matrix, thereby improving the shielding effectiveness. Then, the evenly distributed iron, graphite, and polyurethane matrix also interact strongly to yield a synergistic effect. However, higher iron and graphite content led to reduced tensile and fracture strength, as well as a rougher surface which negatively impacted the filler-matrix interface [218].

Hu et al. synthesized lignin-based polyurethane by in-situ synthesis of modified Fe_3_O_4_, modified CNT, polyethylene glycol and hexamethylene diisocyanate. When the composite contains 10% Fe_3_O_4_, 10% CNTs, and 15% lignin, it exhibits an EMI SE of 37.51 dB [210]. The presence of Fe_3_O_4_ facilitates electron hopping and results in high electrical conductivity in composites. Moreover, the composites demonstrate magnetic loss properties, abundant interfacial polarization, and a well-assembled conductive carbon nanotube network, facilitating efficient absorption, scattering, and reflection of incident radiation. The inclusion of lignin molecules induces a three-dimensional network structure formation with CNTs and Fe_3_O_4_, thereby extending EMW propagation and creating multiple reflection paths within the composites [219]. Additionally, the uniform distribution of CNTs and Fe_3_O_4_ within the polyurethane matrix forms an excellent conductive network, further enhancing electromagnetic shielding effectiveness. Overall, the combined effect of Fe_3_O_4_, CNTs, and lignin significantly enhances the EMI shielding performance of the composite.

Besides, Zhang et al. employed the freeze-drying technique to create a sandwich structure composite with a lignin-based epoxy acrylic ester. The freeze-drying method was used to obtain an intermediate layer by the in-situ reaction of lignin, epoxy resin, acrylic ester, modified Fe_3_O_4_ nanoparticles, and multi-walled CNTs. A 0.5 mm lignin-based epoxy acrylic ester coating was applied on both sides to produce the composite. Notably, when the Fe_3_O_4_ and multi-walled CNT content was at 5% and lignin at 15%, the composite demonstrated an EMI SE of 14.8 dB (Fig. 12a) [220]. The interaction between the benzene rings in lignin and the CNTs facilitates a uniform distribution of CNTs, forming an effective conductive network and improving electromagnetic wave reflection.

In addition, Zeng et al. employed a simple freeze-drying method to prepare a 2 mm thick composite aerogel made of lignin-derived carbon (LDC) and RGO. This aerogel demonstrated an EMI SE of 49.2 dB, featuring three-dimensional, micrometer-sized pores and unidirectional cell walls [126]. The molecular properties of lignin result in an interaction with GO that further leads to RGO/LDC aerogels with thinner, larger, and more cell walls. As a result of tightly packed cell walls, it possesses a larger effective reflective surface area and improves its multiple reflective properties (Fig. 12b). Overall, the combination of numerous reflection effects, high absorption capacity of the cell wall, and a significant number of carrier-induced electrical losses in the cell wall results in the RGO/LDC aerogels with excellent EMI SE at ultra-low density [221, 222]. Besides, Liu et al. incorporated fireproof polypropylene into lignin and combined it with MXene to create functional lignin nanoparticles. This composite material exhibits excellent fire resistance, superior electromagnetic shielding capabilities, and aging resistance (Fig. 12c) [223].

Currently, lignin processing methods are relatively simple and usually involve freeze-drying or in-situ polymerization. Electromagnetic shielding materials typically use Fe_3_O_4_, Fe, and CNT as fillers. By leveraging the porous structure and benzene skeleton of the lignin, Fe_3_O_4_ can regulate electron movement while CNT forms an effective conductive network. The combined effects of these components significantly enhance the EMI SE of the composite [218, 224].

### Bamboo and Its Derivatives

Bamboo represents a green and renewable biomass material with abundant resources, lightweight, easy to process, low cost, short growth cycle and biodegradable [225–227]. Bamboo is composed of bamboo skin, interior and pulp, in which its structural composition may affect the conductivity of different parts [228].

**Fig. 13:**
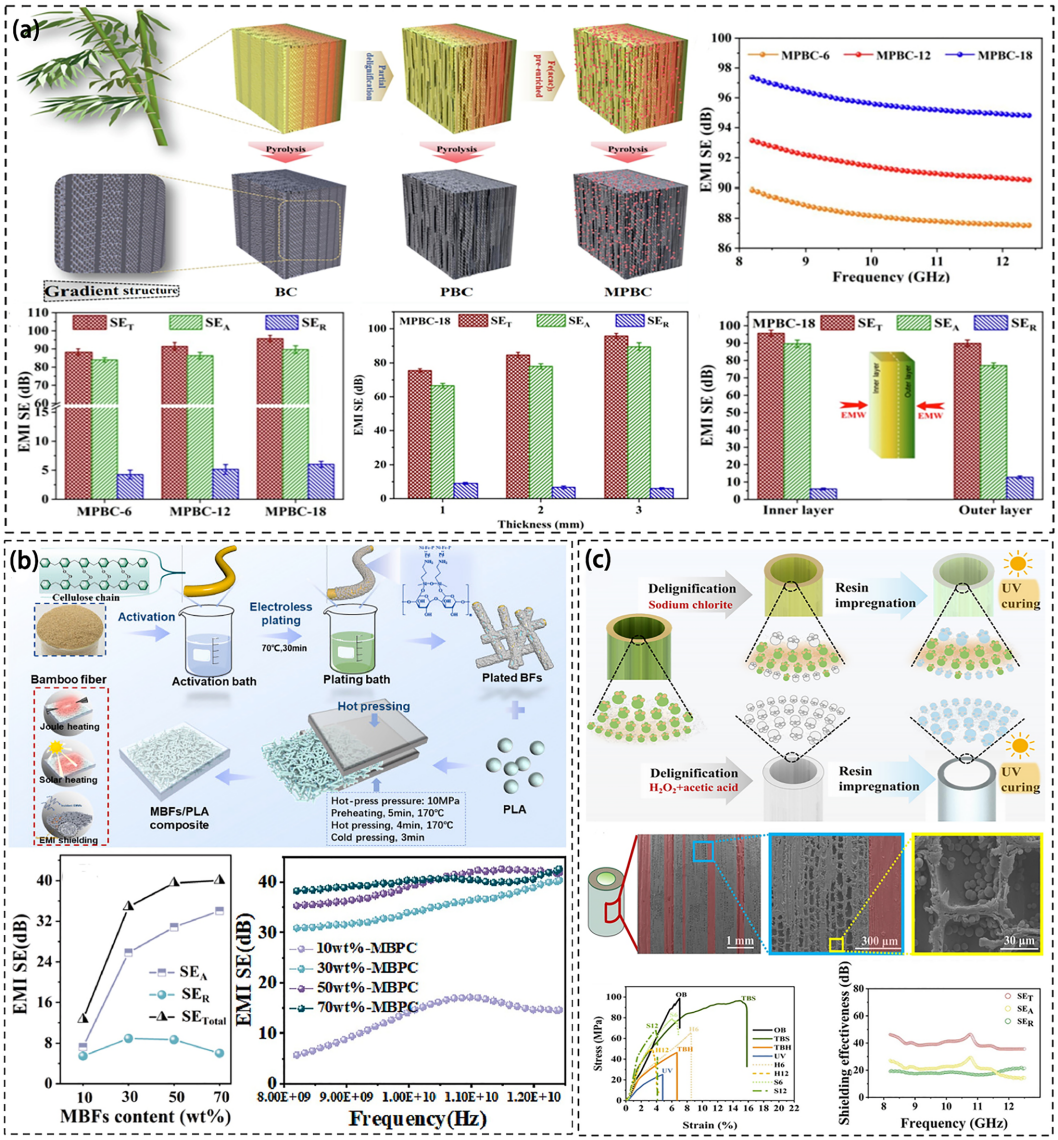
**a** Fabrication process of MBF/PLA composite and EMI shielding performance of MBF/PLA composite [229];** b** Process and principle of nickel activation and surface resistivity of bamboo outer peel, bamboo inner peel, and bamboo pulp [233];** c** Preparation of transparent building bamboo and its mechanical properties and EMI shielding properties [235]

Figure 13 shows the preparation process of bamboo composite materials and the influence of various proportions of concentrations on EMI SE. Zhang et al. performed electroless Ni–Fe-P plating on bamboo fiber as the reinforcement phase. Then, the metallized bamboo fiber was incorporated into polylactic acid (PLA), followed by hot pressing. This resulted in the creation of a metallized bamboo fiber (MBF)/PLA bamboo matrix composite with an EMI SE of 45 dB (Fig. 13a) [229]. The PLA material effectively integrates with the metallized bamboo fiber (MBF) by forming a strong and compatible interface. As the filler content increases, the metal particles distribution on the bamboo becomes more uniform, and the conductive network is improved [230]. The overlapping of MBF within the PLA matrix creates a three-dimensional conductive network that enhances the scattering and electromagnetic losses of the MBF/PLA composite [231, 232]. The use of a higher bamboo fiber (BF) content results in more contact points between the fibers, leading to increased conductive paths and a complete conductive network. Additionally, the metal coating enhances the mechanical strength and thermal stability of the bamboo-based composite.

Zhang et al. coated bamboo with electroless Ni–Fe-P plating [233]. The metal particles are uniformly distributed on the bamboo surface and continuously form a dense conductive network to obtain a uniform and dense metal coating with the crystal structure. For the first time, they studied the difference in the surface conductivity of coated bamboo. The results revealed that bamboo shrinkage rates varied based on vascular bundle density. The adhesion of the metal coating to the bamboo skin was weak, which affected the conductive pathway continuity. Conversely, the metal coating on the bamboo pulp was uniform and dense [92, 234]. The different parts exhibit varying electrical conductivity, with the edge demonstrating higher conductivity than the middle part. This is attributed to the infiltration of metal elements in both the longitudinal and transverse planes (Fig. 13b).

In addition, Wang et al. prepared bamboo-derived carbon (BC) scaffolds with aligned microchannels, layered gradient and anisotropic as shielding materials by pyrolysis, with an EMI SE of 81.52 dB [195]. BC possesses numerous honeycomb pores, which are highly effective for absorbing and shielding EMW. The electrical conductivity of BC is affected by the annealing temperature. As the annealing temperature rises, the outer sheath of BC vascular bundles becomes denser and smoother, leading to increased contraction of vascular bundles and parenchyma, plus a decrease in the cell gap. This results in a reduction in disordered carbon and promotes more uniform grain growth and grain orientation. The abundant ionic motion in BC induces polarization, while the introduction of metal particles enhances electrical conductivity. It was observed that treating BC with lignin increases the number of porous cracks on its surface. A crack-rich surface, gradient porous interior, and excellent conductivity facilitate superior EMW dissipation through multiple internal reflections, relaxation loss, and conductivity loss. Notably, Wang et al. modified bamboo by impregnating UV resin onto the fiber skeleton. This results in a building material with 60% light transmissivity, exceptional mechanical properties, and an EMI SE of 46.3 dB (Fig. 13c) [235].

In summary, electroless plating is primarily utilized in producing bamboo-based materials for electromagnetic shielding. This process capitalizes on the physiological properties of bamboo, resulting in anisotropy and varied conductivity. Bamboo-based composites are characterized by a rich cracked surface, porous interior, and dense conductive network. They also exhibit enhanced electromagnetic wave reflection, thereby achieving superior electromagnetic shielding performance [236].

### Other Biomass Materials

**Fig. 14:**
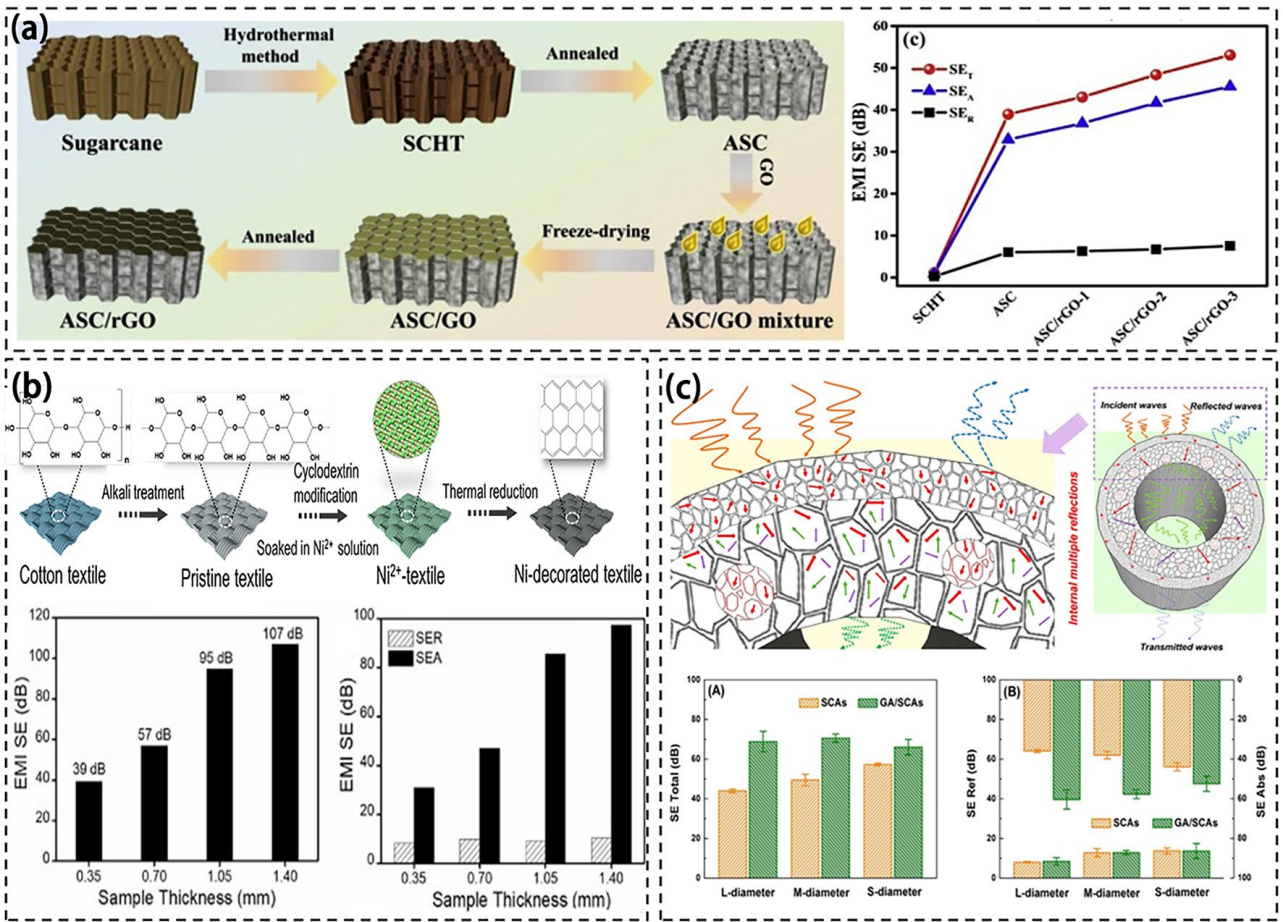
**a** Fabrication process of ASC/RGO and EMI shielding performance of ASC/RGO composite [243];** b** Schematic showing the fabrication process of Ni-decorated textile and EMI shielding performance of Ni-decorated textile [244];** c** EMI shielding mechanism of straw-derived carbon and EMI shielding properties after modification [102]

Figure 14 shows the preparation process the composite materials and their respective EMI SE. Textile and fabrics have been used to prepare EMI shielding materials [237] and other electronics [238–242]. For example, Wang et al. used a hydrothermal reaction to remove lignin from sugarcane and annealed sugarcane (Fig. 14a). The sugarcane/graphene oxide (GO) hybrid foam was obtained by dipping the treated sugarcane into the GO suspension and filling the GO with vacuum-assisted impregnation. The EMI SE of the composite reached 53 dB when the GO content was 17 wt% [243]. The GO was found to be connected to sugarcane through hydrogen bonding and π-π bonding interactions to facilitate electron transfer. Sugarcane cell walls were grafted with many GO nanosheets to enhance the connection within neighboring cell walls and form more conductive pathways. The GO nanosheets were in a closer contact with the increase of GO loading and exhibited stronger electron transport ability. The composite material retains the natural porous structure of sugarcane where its porous structure and rich interfaces reflect and absorb a large amount of EMW, thus improving its EMI shielding performance.

In addition, Peng et al. used Ni as a catalyst to carbonize cotton at 900 °C to produce a textile with an EMI SE of 107 dB [244]. The high degree of graphitization of Ni-treated textiles produced the carbon with a high crystallization degree and a perfect six-membered grid structure, facilitating electron transfer. Ni-treated fabrics with interwoven conductive networks promote the conduction loss in which the dipole polarization is caused by local dipoles on the surface defects of carbon fiber and the end functional groups of affinity agents. The interface polarization is improved with the uniform distribution of Ni particles. Magnetic Ni provides excellent magnetic loss for textiles through eddy current loss, exchange resonance and natural resonance [245]. Multiple EMW scattering and reflections occurred in multilayer micro-structures and nano-structures. These characteristics give nickel-treated textiles excellent electromagnetic shielding properties (Fig. 14b).

Wheat straw has attracted interests [246, 247]. Ma et al. utilized wheat straw to create a structured assembly after carbonization (Fig. 14c). They incorporated ultra-light graphene aerogel into the hollow part of the assembly, resulting in the development of a new electromagnetic shielding material derived from straw. This material exhibits low density and an impressive EMI SE of 66.1 dB [102]. The orderly porous structure of the material allows for multiple EMW reflections within the layers, thereby enhancing its ability to absorb microwaves and ultimately improving its EMI shielding performance.

Existing studies highlight various approaches to fabricate composite materials with significant EMI SE. These methods include utilizing Ni catalysts for carbonization, KOH-activated straw carbon, integrating graphene aerogel into wheat straw, and forming sugarcane/GO hybrid foam. Each technique capitalizes on unique material properties such as a high graphitization degree, porous structures, interconnected pathways, and bonding interactions to optimize electron transfer and enhance EMW absorption. The results emphasize the potential of tailored composite materials to effectively mitigate EMI, thus offering promising solutions across diverse applications [248]. However, cotton straw usually has a high porosity, and its high porosity leads to the leakage of EMWs. Cotton and hemp straw has a large volume, which may affect its use in applications with strict volume requirements such as portable devices. When used in combination with other materials or technologies, cotton and hemp straw still have compatibility issues and requires specific treatment or formulation to ensure shielding effectiveness.

## Electromagnetic Shielding Performance and Application of Biomass Composite Materials

**Fig. 15:**
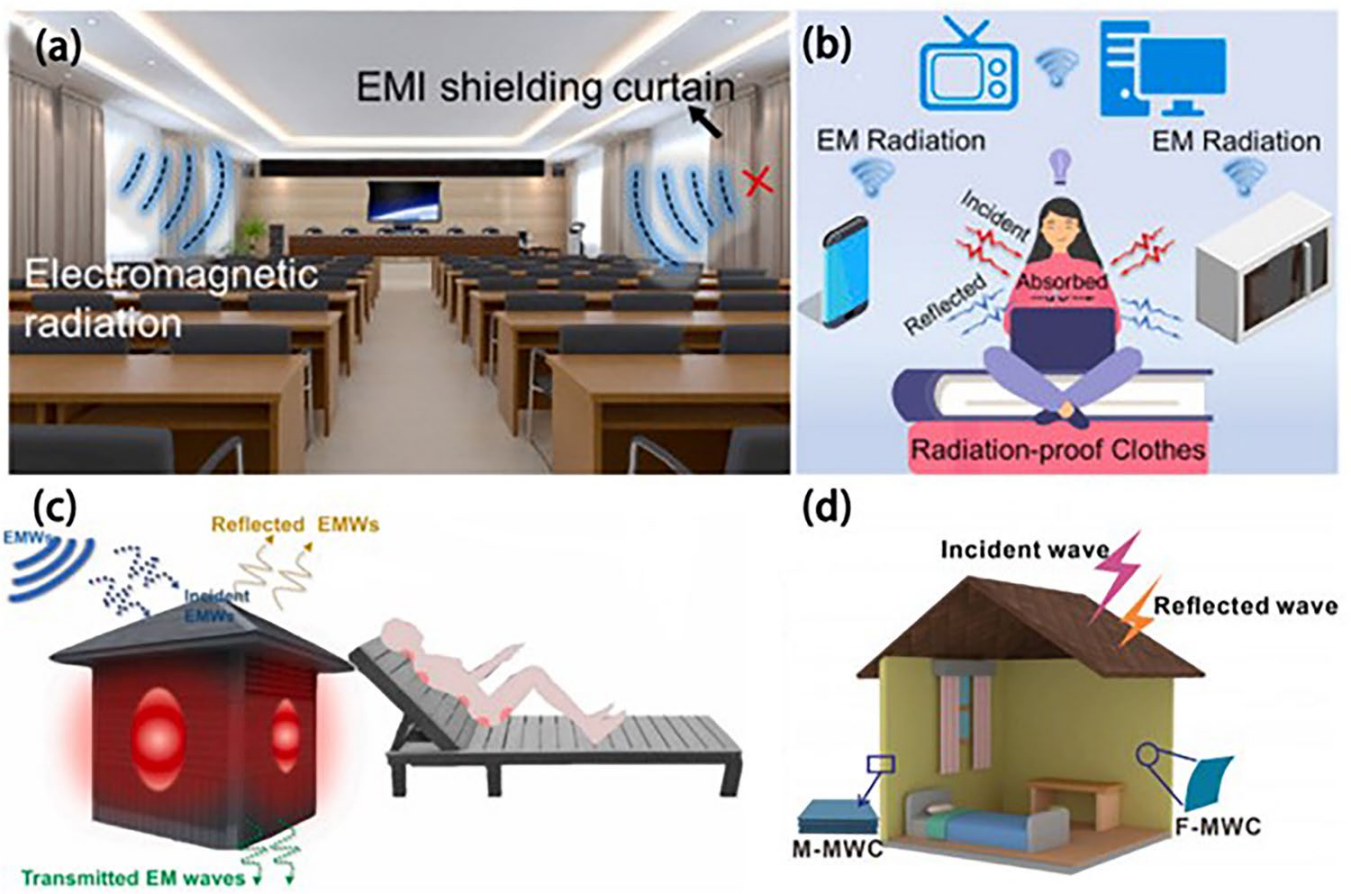
Application of biomass EMI shielding materials in construction, furniture and clothing [145, 229, 244]

MXene/biomass composite materials exhibit impressive mechanical properties, exceptional flame retardancy, and strong electromagnetic shielding capabilities (Table 1). Furthermore, coating treatments can enhance their water and corrosion resistance, making them suitable for use in various challenging environments. These versatile composites find applications in communication, electronics, and residential (Fig. 15a–d). A metal layer is applied to their surfaces through complexation involving metal particles and biomass materials to create a dense conductive network. This results in significantly improved conductivity and electromagnetic shielding properties, which can be attributed to the abundant interfaces and porosity within the composite.

**Table 1:** Conductivity, electromagnetic shielding properties and tensile properties of MXene/Biomass composite materials

MXene/Biomass	Tensile strength (MPa)	EMI SE (dB)	Conductivity (S m^−1^)	Reference
AgNW@MXene/ South American balsa wood	47.8	44.9		[125]
MXene/Cellulose Nanofiber	65.0	53.7	24,875	[200]
WA@MXene/Poplar wood		31.1		[143]
MXene/Balsa wood	68.1	32.7	1858	[145]
MXene/wood-derived hierarchical cellulose scaffold		39.3	6333	[147]

The metal/biomass material is combined to create a consistent metal layer on the surface with a dense conductive network [249]. This composite significantly improves conductivity and electromagnetic shielding properties due to its numerous interfaces and porosity (Table 2). The high hydrophobicity of the metal layer in the wood-based composite electromagnetic shielding material allows the multilayer composite to exhibit excellent hydrophobicity, making it suitable for humid environments. While bamboo-based composites shield slightly less than wood-based composites, they still meet industrial electronic instruments shielding requirements. Considering the unique arrangement of microchannels and superior conductivity of bamboo-based composites, they are well suited to energy storage, conversion, and multifunctional electromagnetic shielding [250]. In short, lignin-based composites are known for their favourable mechanical properties and heat stability, making them a popular option for various military and civilian applications.

**Table 2:** Hydrophobicity, electromagnetic shielding properties and conductivity of composite materials

Metal/Biomass	Hydrophobicity	EMI SE (dB)	Conductivity (S m^−1^)	Reference
Ni/wood/Ni	118.3°	94.1	16.60	[156]
Ni/Cu/wood	123.0°	93.8	29.54	[162]
Ni/wood-derived porous carbon		34.1	9.25	[46]
PLA/MBF		45.0	0.21	[229]
Fe_3_O_4_/Fe/BC		95.6	1238.93	[195]
Ni–Fe–P/ bamboo	119.1°	55.0	4600	[233]

Polymer/biomass materials are known for their relatively thin profile compared to other composites while exhibiting excellent mechanical and conductive properties (Table 3) [84]. These materials possess strong electromagnetic shielding properties that meet commercial use standards. They are frequently utilized in various applications, like electronics, military and civilian fields, robotics, communications, aviation, defense, scalable packaging, and construction materials [17, 251, 252].

**Table 3:** The conductivity, electromagnetic shielding properties and tensile properties of Polymer/Biomass composite materials

Polymer/Biomass	Tensile strength (MPa)	EMI SE (dB)	Conductivity (S m^−1^)	Reference
PEDOT/wood	68.7	46.2	112.8	[167]
PANI/wood		27.6	22.07	[168]
CNT/PU/lignin	7.25	37.5	0.48	[212]
G/PU/lignin	11.7	22.5	0.01	[217]

## Challenges and Prospects

Significant advancements have been achieved in biomass electromagnetic shielding composites in recent years. The growing interest in sustainable EMI shielding composites is attributed to their exceptional properties. However, a dearth of literature on biomass composites for EMI shielding impedes their further advancement. Owing to their remarkable hydrophobicity, flame retardancy and EMI shielding characteristics, these composite materials find potential applications in construction and furniture [253–256], aerospace [257], challenging environments [258, 259], and multifunctional EMI shielding [260, 261].

**Fig. 16:**
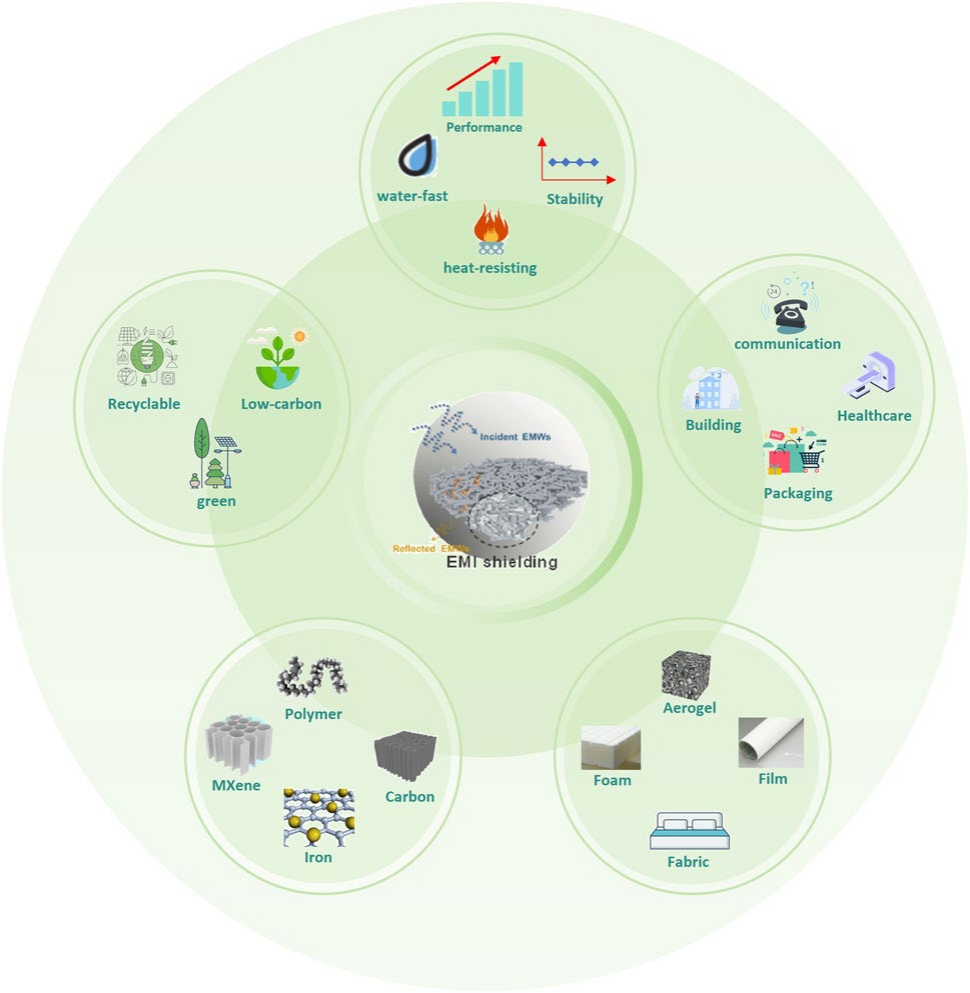
Composite direction, properties, characteristics and applications of biomass EMI shielding materials in the future [229]

Biomass materials can improve their heat resistance through specific treatment and modification, and then maintain a good electromagnetic shielding effect in high temperature environment. The current research landscape in wood gilding predominantly revolves around an electroless nickel or copper plating on wood, with limited exploration of other metals and biomass materials. The EMI shielding and mechanical properties of biomass composites can be enhanced by combining various metals with biomass materials such as silver, aluminum, and iron. In addition, it has a certain impact on the flame-retardant performance of wood, such as adding iron particles, which can catalyze the formation of carbon layers in the combustion process of wood and slow down the combustion inside the wood. Some metal particles may also form a protective layer of metal oxide on the surface of the wood to achieve flame retardant effect. The existing methods for synthesizing wood composites are complex and time-consuming. Furthermore, the poor interfacial compatibility of wood with other materials coupled with its inherent defects including anisotropic and inhomogeneous characteristics, wet swelling, dry shrinkage, and susceptibility to corrosion, could significantly impact electromagnetic shielding performance [168, 262]. As such, prospective research endeavors could commence with chemical modification and surface treatment of wood to obtain a uniform surface coating and a dense conductive network (Fig. 16).

Most treatment methods for cellulose materials are focused on surface treatment or modification, with a particular attention to the impact of three-dimensional pores on electromagnetic shielding. The resulting composite exhibits favorable mechanical properties and lightweight characteristics and significantly surpasses commercial requirements for EMI SE. However, limited research has been conducted on its waterproofing, heat resistance, and mildew resistance properties. This makes it impossible to use in wearable, portable designs. Cellulose materials can be used to construct multifunctional EMI shielding materials with flame retardancy by in-situ polymerization and coating technology. In addition, by combining with other nano-fillers, such as metal nanoparticles, not only the EMI SE can be improved, but also the flame retardant performance can be enhanced. The flame retardant properties of cellulose-based materials can improve the safety of electromagnetic shielding materials, especially in environments with dense electronic devices and high power applications. Moreover, it can have a wider application potential in aerospace, military equipment, electronic products and other fields with high safety requirements. Future developments in cellulose materials should prioritize performance optimization to ensure reliable functionality under challenging environmental conditions.

Lignin-based composites may exhibit diminished mechanical properties in terms of elongation at break and reduced tensile strength, which could be attributed to metallic elements and graphite. Consequently, the EMI SE of these composites is inferior to that of their wood-based counterparts, with some failing to meet commercialization standards. In order to enhance their electromagnetic shielding performance, the composites have been augmented with fillers such as Fe_3_O_4_, Fe, G, and CNT. Optimal electromagnetic shielding could be achieved by adjusting the lignin, iron powder, and graphite ratios. Nonetheless, these studies overlooked flame retardancy, hydrophobicity, and mildew resistance [212]. In order to overcome these constraints, forthcoming research endeavors should prioritize enhancements in the mechanical properties of composites, the chemical modification of lignin to instill advantageous chemical attributes, and the expansion of potential applications for lignin-based composites.

The heterogeneous physiological structure of bamboo results in varying coating thickness across its parts, leading to differences in resistivity and shielding effectiveness [263]. For future research endeavors, it is recommended that bamboo undergoes pretreatment to mitigate the influence of its structure on its functionality. This may involve acid treatment, alkali treatment, and physical modification to improve the mechanical and physical properties of bamboo, enhance dimensional stability, and augment its compatibility with the polymer matrix [264, 265].

In addition, future research in biomass composites should be on developing multifunctional biomass EMI shielding composites that can be effectively utilized in practical applications. It is essential that these composites not only exhibit outstanding electromagnetic shielding properties but also demonstrate remarkable environmental adaptability, including weather resistance, Flame retardancy, corrosion resistance, and waterproof properties. There is still significant potential for innovation and advancement in the research of biomass EMI shielding composites, particularly in enhancing electromagnetic shielding materials to be lightweight and thin, while possessing multifunctional attributes.

## Conclusions

The increasing prevalence of electromagnetic pollution posits a considerable intimidation to information security, ecosystems and human health. As environmental consciousness grows, and resources are increasingly depleted, traditional electromagnetic shielding materials have revealed their inherent limitations in research and production. Consequently, the development of environmentally friendly and sustainable EMI shielding materials is crucial for safeguarding electronic equipment, preventing information leakage, and protecting public health.

This paper summarizes recent developments in biomass EMI shielding composites, including the underlying mechanisms of EMI shielding, the applications of various types of biomass EMI shielding materials, and the preparation methods for biomass EMI shielding composites such as coating, impregnation, in-situ polymerization, in-situ insertion, and chemical plating. Mainstream processing methods namely MXene composite and chemical plating, have been instrumental in enhancing the shielding performance, mechanical properties, and flame retardant capabilities of these composites. Additionally, modifications to biomass materials have induced desirable properties such as transparency, waterproofing, and mildew resistance, thereby expanding the potential applications of biomass materials in electromagnetic shielding. Despite these advancements, research on biomass EMI shielding composite materials remains relatively limited compared to other materials. Several unresolved issues persist involving inherent defects in biomass materials, anisotropy, varying electrical conductivities in different parts, corrosiveness, single functionality, and the non-waterproof nature of biomass materials.

1. *Diversity of materials* Biomass materials are limited for EMI shielding, with wood being the primary raw material for modification and relatively simple reagents being used for the modification process. However, certain wood properties, such as corrosion resistance, heat resistance, and hydrophobicity, do not meet the requirements. Additionally, wood modification often produces toxic gases. Therefore, it is essential to comprehensively compare various biomass materials and select those that meet the requirements for modification or composite. This approach can significantly reduce production costs and environmental pollution. The use of different fillers for treatment may yield varying effects such as the production of transparent wood resistant to EMI and bamboo with uniform inner and outer layer properties. By comparing a range of biomass materials, it is feasible to obtain satisfactory chemical and mechanical properties that meet commercial standards. Thus, increasing the diversity of biomass materials is crucial as it creates electromagnetic shielding materials with special properties through modification or composite production to address the specific needs of consumers.
2. *Net zero emissions* In the production process of biomass materials, it is imperative to consider the modification of different fillers as it can release harmful gases. These harmful gases can be reduced by simplifying the experimental steps or reselecting the appropriate fillers. It is also crucial to assess the potential generation of greenhouse gases throughout the entire life cycle including the source, production, sales, and recycling stages to devise promising strategies for emission reduction. A comprehensive life cycle approach could be established to achieve zero carbon emissions. In order to meet commercial requirements, efforts should be made to improve production efficiency, ensure pollution-free production, facilitate convenient recycling, and further reduce greenhouse gas emissions and environmental pollution.
3. *Machine learning* Through the analysis and research of biomass materials, various fillers can be analyzed through artificial intelligence (AI) technology to improve the performance of biomass materials and improve one or more mechanical or chemical properties. Through machine learning, more suitable preparation processes or equipment can be selected to meet the high efficiency, safety and reliability of production. Through machine learning and AI technology, the performance and production efficiency of materials can be greatly improved, and the danger of experiments can be avoided, and environmental pollution and greenhouse gas emissions can be reduced.
4. *Circular economy* The utilization of biomass materials as the primary components for electromagnetic shielding materials offers several advantages. These materials are readily available from natural sources and can be extricated from wastepaper or other products, hence providing sustainable secondary uses. From a life cycle perspective, this approach significantly extends the lifespan of the materials and reduces waste. Waste can be recycled multiple times for pulp molding or padding and subsequently for versatile applications. Maximizing the utilization of biomass materials across the entire lifecycle, from sourcing and production to sales and recycling, a high raw material utilization rate can be achieved to increase natural recycling, thereby mitigating environmental impact.
